# Poly(lactic acid) hybrid nanocomposites with synergistic cellulose nanocrystal-carbon reinforcement for sustainable packaging and multifunctional applications

**DOI:** 10.1039/d6ra05823j

**Published:** 2026-09-16

**Authors:** Kartikey Verma, Salim H. Siddiki, Chandan Kumar Maity, Bhuvaneshwari Balasubramaniam, Anup Singh, Tiyasha Tiyasha, Suraj Kumar Bhagat, Ankush Mehta, Jitendra Bahadur

**Affiliations:** a Krishna Institute of Engineering and Technology (KIET) Ghaziabad Delhi-NCR Uttar Pradesh 201206 India; b Department of Chemistry and Chemical Biology, IIT (ISM) Dhanbad 826004 Dhanbad India; c Department of Chemistry, Gachon University 1342 Seongnam-daero, Sujeong-gu Seongnam-si Gyeonggi-do 13120 Republic of Korea ckmkorea@gachon.ac.kr; d Department of Chemistry, Indian Institute of Technology (BHU) Varanasi Varanasi Uttar Pradesh 221005 India; e Directorate of Research, Innovation and Consultancy (DRIC), Chandigarh University Uttar Pradesh Unnao 209859 India; f Department of Civil Engineering, School of Engineering and Computing, Dev Bhoomi Uttarakhand University Dehradun India; g Centre for Interdisciplinary Research, SRM University-AP Amarawati Andhra Pradesh 522240 India surajkumar.b@srmap.edu.in; h School of Engineering and Technology, K. R. Mangalam University Gurugram 122103 Haryana India; i Electronics and Communication Engineering, Amrita School of Engineering, Amrita Vishwa Vidyapeetham Bengaluru 560103 Karnataka India

## Abstract

Sustainable and high-performance polymer composites are needed for advanced technological applications. This study outlines the straightforward synthesis and comprehensive analysis of novel bio-nanocomposites based on poly(lactic acid) (PLA) that are reinforced in a combined way with cellulose nanocrystals (CNC) and a hybrid filler system comprising carbon nanotubes (CNTs) and reduced graphene oxide (rGO). The addition of CNTs and rGO resulted in improved crystallinity. FTIR analysis indicated changes in the local chemical environment consistent with noncovalent matrix–nanofiller interactions. Differential Scanning Calorimetry (DSC) demonstrated that 0.50 wt% rGO exhibited the most favorable thermal behavior, as well as significantly enhanced crystallinity. Thermogravimetric analysis (TGA) demonstrated that nanocomposites exhibited enhanced thermal stability by the incorporation of nanofillers. The contact angle rising from 56.2° in PLA/CNC to 82.9° in 1 wt% rGO loading showed enhancement in hydrophobicity. Lysozyme-assisted degradation tests revealed slower degradation rates in composites loaded with nanofillers, which indicates improved resistance to enzymatic breakdown. A substantial decrease in water vapor permeability (WVP) and oxygen permeability (OP) study revealed improving barrier characteristics. The Alamar blue assay was carried out to study the cytotoxicity of hMSCs (human mesenchymal stem cells). The 0.25 wt% CNT-loaded composite showed a highly significant increase in cell survival by day 7 (*p* < 0.001). This study demonstrates a route for developing PLA nanocomposites with tunable structural, thermal, barrier, surface, and degradation properties.

## Abstract 2

Sustainable and high-performance polymer composites are needed for advanced technological applications.

## Graphical abstract

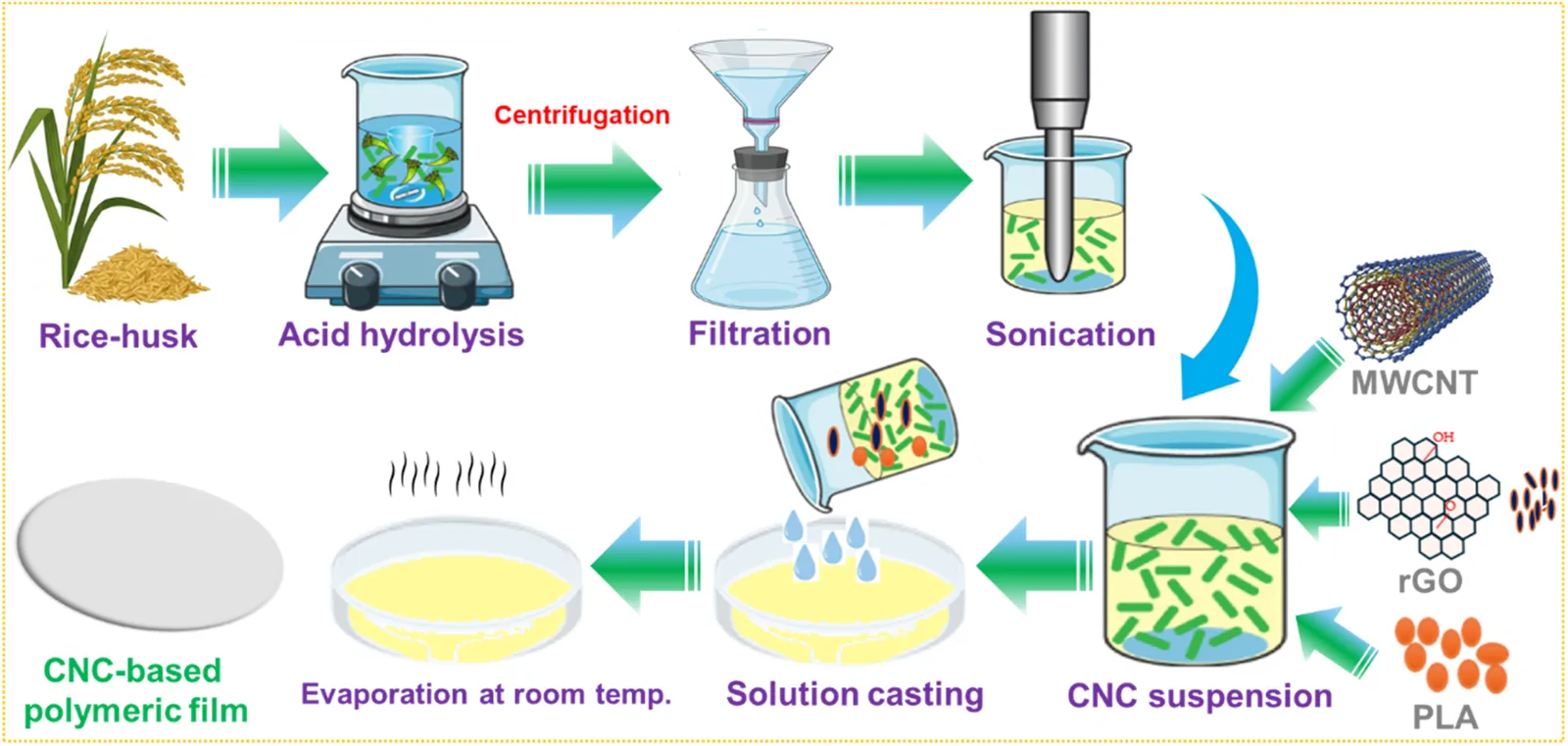

## 1. Introduction

Biopolymers are increasingly being recognized as viable substitutes for conventional petroleum-derived plastics. Research has been gradually shifted towards the synthesis of robust and lightweight biodegradable polymer nanocomposites. In this respect, PLA produced from renewable sources like corn starch turns out to be a promising material for packaging applications. PLA still suffers from inherent limitations, including poor thermal stability and low melting strength, which limit its broader application.^1–5^ PLA also possesses certain characteristics such as low heat and impact resistance, poor melting rate, sensitivity to hydrolysis, a sluggish crystallization rate, and gas permeability, which restrict its usage in advanced applications.^6–9^ To overcome these limitations, significant effort has been made to address PLA's shortcomings. Incorporation of nanomaterials like graphene, nanoclay, and CNTs as nanofillers into polymer matrix composites has improved the physico-chemical properties of the composites compared to the host polymer.^10–12^ The mechanical properties of polymer nanocomposites depend on effective matrix–filler interactions and uniform nanofiller dispersion. Achieving both simultaneously, however, remains a considerable challenge due to the inherently high surface energy of nanofillers and their tendency to aggregate in hydrophobic environments.^13^ Achieving uniform nanofiller dispersion and effective matrix–filler integration remains challenging because of the strong tendency of nanoscale fillers to aggregate within polymer matrices. Appropriate filler selection, concentration, and processing conditions are therefore important for obtaining homogeneous composites with improved functional properties. Among various bio-based reinforcements, CNCs have been widely investigated as biodegradable fillers in the PLA matrix owing to their renewable origin, nanoscale dimensions, and ability to significantly enhance the properties of polymer composites.^14–19^ This is mostly due to the biodegradability, renewability, and non-toxicity properties of CNCs. They possess not only low density but also outstanding mechanical strength and stiffness (modulus 130–200 GPa and tensile strengths 2–3 GPa), as well as a significant surface area. This would increase the number of spherulites that crystallize and cause a decrease in crystal size.^20–24^ The incorporation of CNCs into PLA leads to remarkable enhancements in both modulus and tensile strength compared to neat PLA; however, certain challenges remain to be addressed. In many cases, the thermal and mechanical properties of the polymer matrix have been found inadequate in comparison to the theoretical predictions due to the apparent aggregation of CNCs and poor nano-dispersibility.^25–27^ Adding CNCs to composites usually makes them more brittle since the hydrophobic polymer matrix doesn't interact well with the CNCs. To overcome this problem, carbon materials like CNT and rGO have been incorporated because they show high strength, rigidity, electrical conductivity, and high surface area.^28–30^ CNTs improve the atomic structure and mechanical characteristics of the resulting material, making them very beneficial fillers.^30^ The interfacial contact between carboxyl-functionalized CNTs and PLA seems to be increased by surface functionalization.^30^ The extraordinary physical, thermal, optical, and electrical properties of rGO have led to its use as an additional nanofiller.^31–34^ Reinforcing at extremely low loading levels, it can substantially improve the mechanical characteristics of the host polymer as an additional nanofiller. CNCs and CNT/rGO are incorporated into the polymer to improve the rigidity, durability, and stiffness of PLA nanocomposites, as well as to achieve interfacial adhesion and optimal dispersion between the polymer matrix and nanofillers.

In this work, we synthesised CNCs from rice husk and then prepared PLA composites with 1 wt% CNC. 1 wt% CNC is the most suitable concentration in the PLA matrix, as higher CNC concentrations agglomerated and dramatically reduced mechanical and barrier properties, negating the benefits of the reinforcement.^35^ The incorporation of 1 wt% CNC into the PLA matrix was selected as the reference, since numerous prior reports have already established the behavior of pristine PLA and PLA/CNC composites.^29,33^ To improve the robustness of the nanocomposites, additional nanofillers, CNT and rGO with varying concentrations, were incorporated into the PLA/CNC nanocomposite, and a comparison was made between the two nanofillers. The toxicity, biocompatibility, and barrier properties of the synthesised samples were also investigated through cytocompatibility studies, OP, and WVP analysis. This study was undertaken to investigate the degradation of PLA modified with CNC/CNT/rGO, in light of the importance of the biodegradation of PLA and the inconsistency of the available data. The developed PLA/CNC/CNT/rGO films are designed to combine moisture and oxygen barrier protection with enhanced thermal stability, tunable surface wettability, and controlled degradation, thereby providing multiple functions within a single biodegradable material.

## 2. Experimental section

All analytical-grade chemicals were used for the synthesis of composites. The chemical details are discussed in Section S1.1 (SI).

### 2.1 Synthesis of CNC from rice husk

Rice husk was cleaned with clean water first, then rinsed with deionized water to eliminate remaining surface contaminants and soil particles. After being cleaned and dried in an oven at 60 °C for 30 hours, rice husk was changed into powder form with a grinder to achieve a uniform particle size. 20 g of rice husk powder was soaked in 500 mL of 4% NaOH and stirred for 2.5 hours at 80 °C to remove the lignin. After filtering the suspension, any remaining residue was rinsed with deionized water until a neutral pH was obtained. Since some black color was present, the process was repeated once to remove the hemicellulose and lignin. Using a solution of 1.7% (w/v) sodium chlorite (NaClO_2_), pH 4.5, which was adjusted with acetic acid, the residue was taken through the bleaching process. The procedure was conducted for 2 hours with continuous stirring at 75 °C. This was done twice more until the residue became completely white, which indicated that the lignin had been removed successfully.^36^ The residue was thoroughly cleaned with deionized water first and then dried at 60 °C in a hot-air oven. Bleached cellulose (residue) was then dispersed in 100 mL of sulfuric acid (64 wt%) and stirred at 45 °C for 1 hour. The quenching process was carried out by dilution with 500 mL of cold distilled water to stop the reaction immediately. Excess acid was removed from the suspension by centrifuging it at 10 000 rpm for 20 minutes. This procedure was carried out repeatedly until the suspension was almost neutral (pH = 7). To obtain a constant neutral pH, the acid-washed suspension was dialyzed with distilled water for 5 days using dialysis tubing. To ensure uniform dispersion of the nanocrystals and break down any agglomerates, the dialyzed CNC solution was then sonicated for 15 minutes in an ice bath using a probe sonicator. CNC suspension was then freeze-dried and stored for further use.

### 2.2 Preparation of PLA/CNC/CNT/rGO composites

**Fig. 1:**
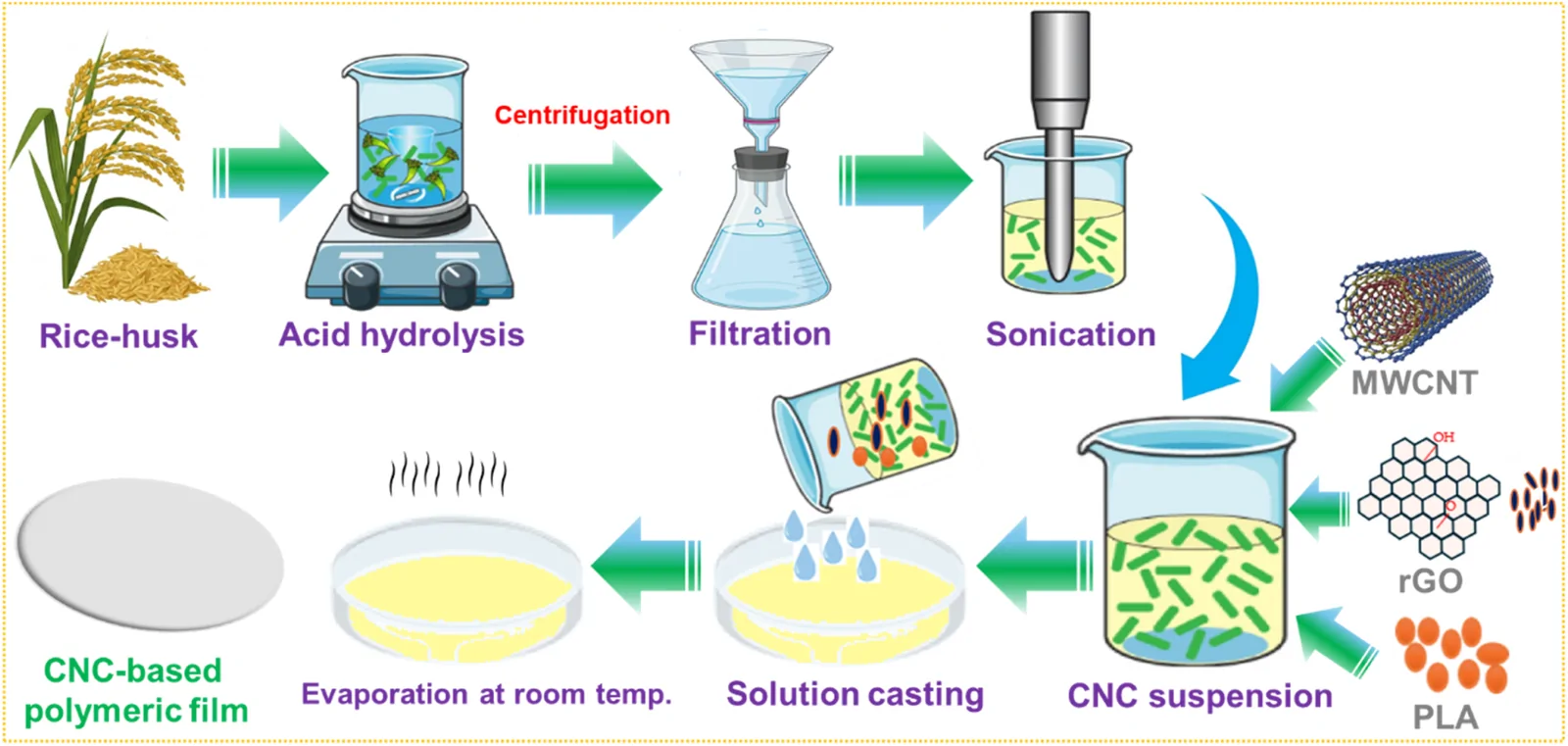
Schematic diagram for CNC extraction from rice husk and synthesis of PLA/CNC/CNT/rGO nanocomposites through the solution casting process.

Initially, graphene oxide (GO) was synthesized through a well-established modified Hummers' method.^37^ For the synthesis of rGO, GO was chemically reduced with hydrazine hydrate (N_2_H_4_·H_2_O). The detailed synthesis procedure of rGO from GO is discussed in Section S1.2 (SI). The PLA nanocomposites were synthesized using the solution casting technique. First, CNC was dispersed in deionized water, and to prevent aggregation and ensure full dispersion, probe sonication in an ice bath was carried out for 30 minutes. After this, solvent exchange was performed to transfer CNC into chloroform. PLA granules were dissolved in chloroform at 5 wt% (w/v) in a separate container for 12 hours under constant stirring at 35 °C until complete dissolution was achieved. Dispersed 1 wt% CNC were then added drop by drop into the PLA solution. To ensure that CNCs were evenly mixed throughout the polymer solution, the mixture was constantly stirred for 24 hours at 35 °C. rGO and CNT with different concentrations were dispersed in chloroform at a concentration of 0.05% (w/v) of chloroform. For one hour, the dispersion was carried out with a tip sonicator to prevent agglomeration. Nanofiller dispersions (CNT and rGO) were added into PLA + 1 wt% CNC solution under constant stirring for 3 hours. To improve the dispersion of the nanofiller within the PLA and make it uniform, ultrasonication (bath sonicator) was carried out for 20 minutes. After that, the homogeneous solution was transferred into a glass Petri dish. All films were dried at 35 °C temperature for 72 hours for the removal of trapped chloroform. To fully remove the solvents, the films were kept to dry for 24 hours at 45 °C in a vacuum oven. Fig. 1 shows the schematic diagram for the synthesis of PLA/CNT/CNC/rGO-based nanocomposites by the solution casting method. Table 1 summarizes the sample codes and the amount of different components used for synthesizing PLA/CNC/CNT/rGO nanocomposites.

**Table 1:** Sample code and the number of different components used for PLA/CNC/CNT/rGO nanocomposites

Sample code	PLA (wt%)	CNC (wt%)	CNT (wt%)	rGO (wt%)
Sample 1	99.0	1.0	—	—
Sample 2	98.75	1.0	0.25	—
Sample 3	98.75	1.0	—	0.25
Sample 4	98.5	1.0	0.50	—
Sample 5	98.5	1.0	—	0.50
Sample 6	98.0	1.0	—	1.0

The films were prepared at laboratory scale by solution casting on glass Petri dishes. Film thickness and thickness uniformity were not quantitatively determined in the present study.

The material characterization techniques and all experimental procedures are vividly discussed in S1.3 (SI).

## 3. Results and discussions

### 3.1 Structural, morphological, and thermal analysis

**Fig. 2:**
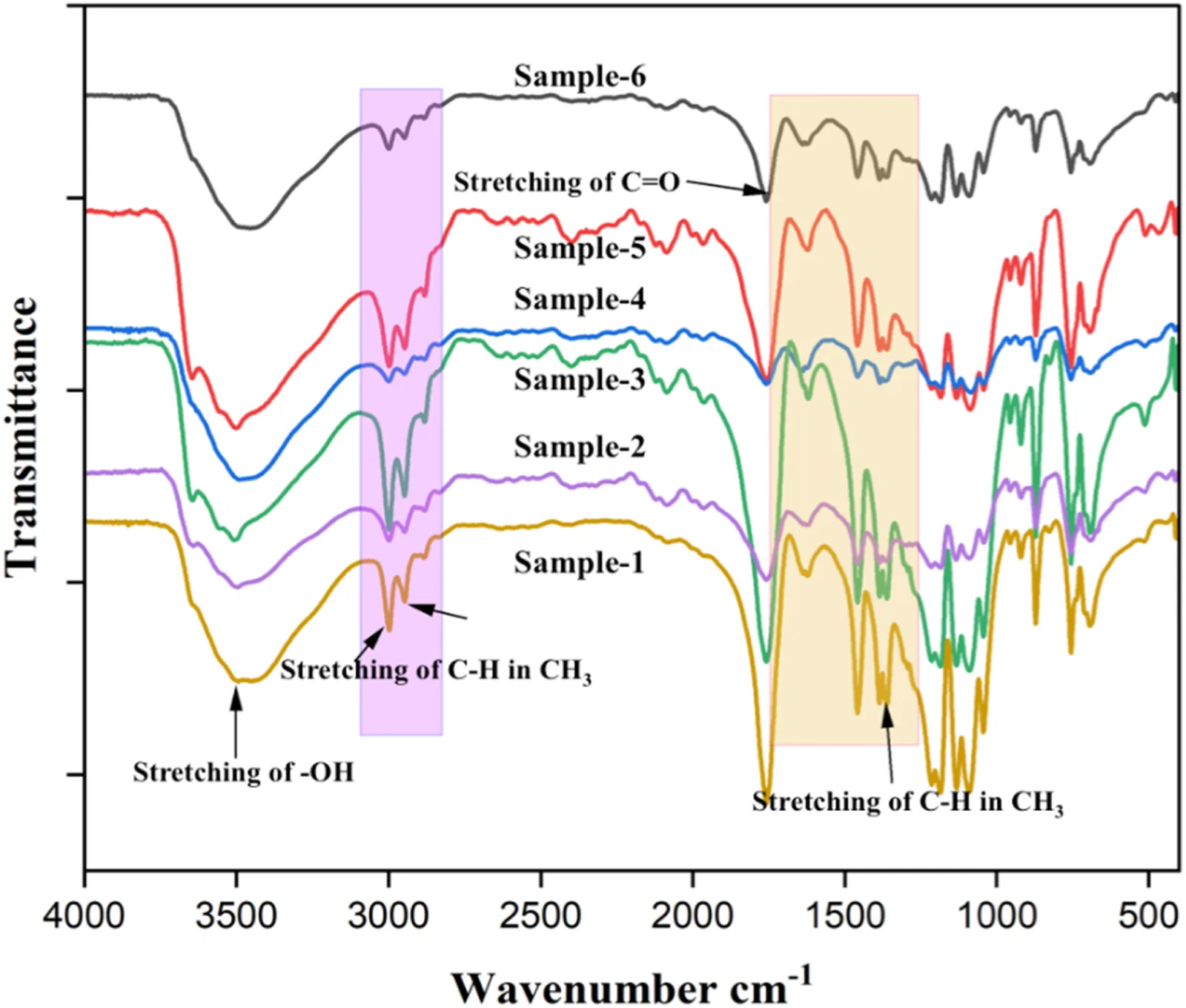
FTIR spectra of PLA/CNC nanocomposite films with varying concentrations of CNT and rGO.

Fourier Transform Infrared (FTIR) spectroscopy was done to investigate the chemical structure and intermolecular interactions within the polylactic acid (PLA)/cellulose nanocrystal (CNC) composites with varying concentrations of CNT and rGO. The spectra for all composite samples are presented in Fig. 2. Sample 1 (PLA/1 wt% CNC), taken as a reference sample, displays the characteristic absorption bands of the PLA matrix. The peak observed at 1755 cm^−1^ is the most prominent peak, which is sharp & intense. This peak is attributed to the stretching vibration of the carbonyl group (C=O) in the ester linkage of PLA. The peaks at 2995 cm^−1^ correspond to the asymmetric stretching vibration, and the peak at 2946 cm^−1^ corresponds to the symmetric stretching vibrations of the methyl groups (–CH_3_ and –CH) in the PLA backbone.^34^ The strong peaks observed in the 1000–1300 cm^−1^ region are attributed to C–O–C stretching vibrations of ester linkages. The peaks at 1453 cm^−1^ and 1362 cm^−1^ correspond to –CH bending vibrations. A broad absorption band centered around 3467 cm^−1^ is also visible, which can be attributed to the –OH stretching vibrations from the terminal hydroxyl groups of the PLA chains and, more significantly, from the abundant –OH groups present on the surface of the incorporated CNCs. Upon the introduction of CNTs (sample 2 and sample 4) and rGO (sample 3, sample 5, and sample 6), the resulting spectra show a similarity to the reference PLA/CNC spectrum. No new significant absorption peaks corresponding to CNT or rGO are observed. This is expected due to the low concentration, as the weight percentage of CNTs and rGO is very low (0.25 wt% to 1.0 wt%), so their characteristic signals are likely covered by the strong absorption bands of the PLA/CNC matrix, which constitutes the bulk of the material. The fundamental vibrations of the graphitic backbone (C=C) in pristine CNTs and perfectly reduced graphene are infrared-inactive due to a lack of a net dipole moment change. While some weak signals can arise from residual functional groups on rGO (*e.g.*, carboxyl C=O at ∼1720 cm^−1^ or hydroxyl O–H at ∼3400 cm^−1^) or defects, they are very weak. For sample 5, enhanced peak broadening in the fingerprint region (600–1200 cm^−1^) is linked to PLA chain distortion or reduced crystallinity due to intercalation of rGO nanosheets. Broadening and shift of the ester C=O peak (1753 cm^−1^) in sample 6 support strong matrix–nanofiller interaction and possible reduction in polymer chain mobility. The absence of any other noticeable shifts or the appearance of new bands indicates that the addition of CNTs and rGO does not lead to the formation of new covalent chemical bonds with the PLA or CNC components. The absence of new absorption bands or significant peak shifts indicates that CNT and rGO are incorporated into the PLA/CNC matrix without forming new covalent bonds. The observed broadening and slight shift of the carbonyl band, particularly at higher rGO loading, suggest changes in the local polymer environment and matrix–nanofiller interactions. These interactions are therefore attributed primarily to noncovalent forces, including van der Waals interactions and hydrogen bonding involving the polar groups of PLA/CNC and residual oxygen-containing groups on rGO. Together with the SEM and XRD results, these observations support effective physical incorporation and dispersion of the nanofillers within the PLA/CNC matrix.

**Fig. 3:**
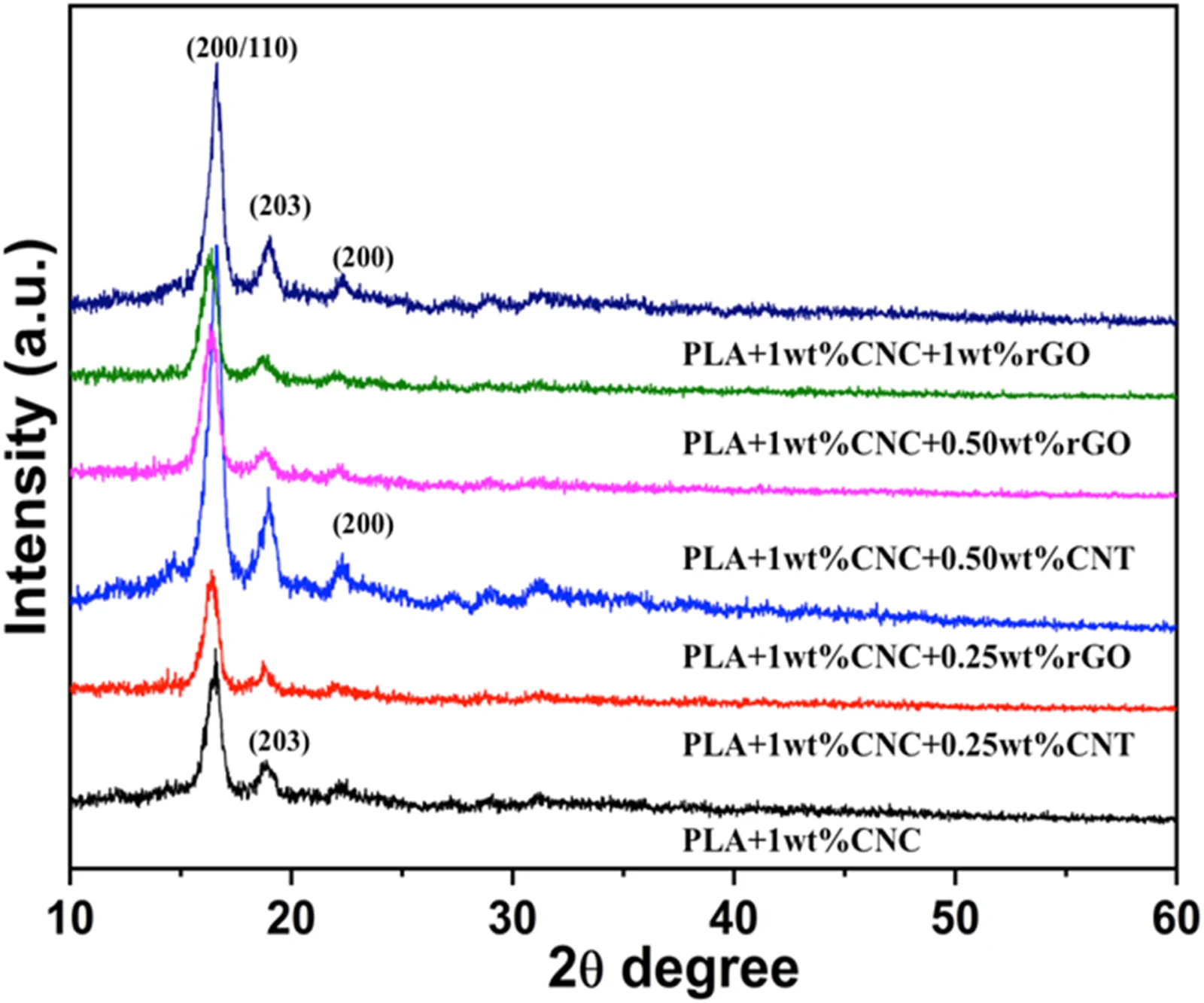
XRD patterns of PLA/CNC nanocomposite films containing varying concentrations of CNT and rGO nanofillers.

**Fig. 4:**
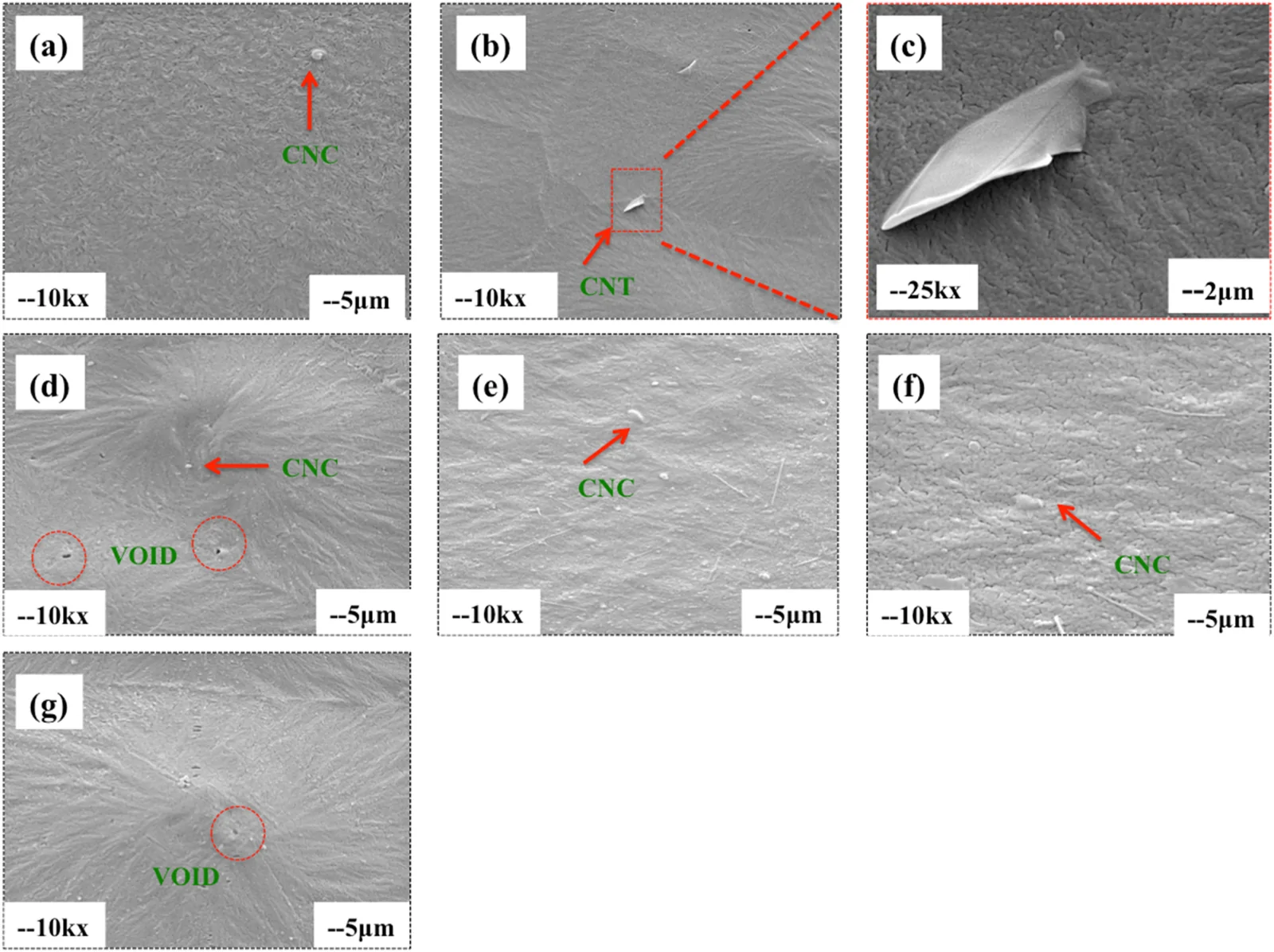
(a–g) SEM images of fractured surfaces of PLA/CNC-based nanocomposites.

The XRD patterns of PLA-based nanocomposites reinforced with 1 wt% CNC and varying concentrations of CNT and rGO are shown in Fig. 3. The analysis presents an in-depth understanding of the impact of nanofiller type and concentration on the crystalline structure of the PLA matrix. The diffraction pattern of the reference (sample 1) establishes the baseline structural characteristics. A semi-crystalline structure is suggested by the presence of numerous well-defined Bragg reflections. The primary reflection, located at 2*θ* = 16.63°, is a composite peak corresponding to the (200)/(110) crystallographic planes of the α-form of PLA.^38^ Secondary, less intense reflections are observed at 2*θ* = 18.9° corresponding to the (203) plane and a broad feature around 2*θ* = 22.4°. This peak corresponds to the (200) plane of the cellulose I from the CNCs. The peak profile of the reference sample represents the intrinsic crystallization behavior of the PLA/CNC system under the utilized processing conditions. Upon introduction of 0.25 wt% CNT (sample 2), a subtle but distinct sharpening of the principal (200)/(110) peak at 16.59° is observed relative to the reference. The (200)/(110) peak at 16.63° in sample 3 is visibly the most intense and sharpest among all samples. The increased intensity and sharpening of the PLA reflection are consistent with enhanced crystallization in the presence of rGO. The high surface area of CNTs and the planar structure of rGO may provide heterogeneous nucleation sites for PLA crystallization. A similar trend is evident for the rGO-reinforced composites. In sample 4 (0.50 wt% CNT), the intensity of the 16.61° peak is noticeably greater than that of the reference (sample 1), confirming the nucleating ability of CNT even at low loading. Increasing the concentration to 0.50 wt% rGO (sample 5) and further to 1 wt% (sample 6) results in an increase in the intensity of this primary peak. This shows that, like CNTs, rGO's planar surfaces act as a pattern for PLA crystallization. This enhancement with concentration highlights a clear dose-dependent relationship between the rGO content and the degree of crystallinity in the PLA matrix. A qualitative comparison between sample 4 (0.50% CNT) and sample 5 (0.50% rGO) suggests that CNTs may impart a slightly stronger nucleating effect at equivalent weight fractions, as evidenced by the sharper peak in the sample 4 spectra. A critical observation across all nanocomposite spectra (samples 2–6) is the complete absence of the characteristic diffraction peak at 2*θ* = 26° for the (022) plane of stacked graphitic structures. This confirms that the CNT and rGO are homogeneously distributed throughout the PLA/CNC matrix. The absence of a distinct diffraction feature near 2*θ* ≈ 26° suggests that pronounced graphitic restacking is not evident in the composite films. The dispersion of CNT and rGO was further assessed from the SEM morphology (Fig. 4). In conclusion, the addition of nanofillers increased the crystallinity and, by extension, altered the properties of the materials. The diffraction peaks became sharper and more intense, resulting in a decrease in the FWHM. The enhanced crystallinity often translates to improved mechanical strength and thermal stability of the composite material. Table 2 summarizes Crystallinity Index (% CI), *d*-spacing (Å), and Full Width at Half Maximum (FWHM) for the major PLA crystalline peak near 2*θ* ≈ 16.2°, extracted from the XRD pattern.

**Table 2:** Structural parameters derived from XRD patterns for the PLA + CNC nanocomposites. Analysis focuses on the principal (200)/(110) reflection

Sample	2*θ* (°)	*d*-Spacing (Å)	FWHM (°)	Relative crystallinity index (% CI)
Sample 1	16.62	5.29	0.89	29.6
Sample 2	16.59	5.27	0.81	32.9
Sample 3	16.63	5.27	0.83	37.2
Sample 4	16.61	5.28	0.73	36.6
Sample 5	16.65	5.30	0.75	40.4
Sample 6	16.6 3	5.30	0.72	44.1

The SEM images demonstrate the morphological development of the fractured surface of PLA-based nanocomposites upon the incorporation of CNC, CNT, and rGO fillers. Fig. 4a, the reference sample (sample 1) showed a comparatively homogeneous and smooth surface, suggesting that the CNCs are well distributed throughout the PLA matrix. The CNC particles are well dispersed and incorporated, suggesting uniform dispersion and strong interfacial adhesion. At low CNT loading, the fractured surface appears relatively smooth with distinct CNT incorporation (Fig. 4b). This is further confirmed by the enlarged view (Fig. 4c), which shows a platelet-like morphology within the polymer matrix. This indicates partial alignment and mechanical interlocking of CNTs, which can improve load transfer. Fig. 4d shows that surface roughness starts increasing as rGO is used as a nanofiller. Distinct voids (highlighted in the figure) and radial crack patterns are also observed, which arise from solvent evaporation during film drying at room temperature.^38^Fig. 4e shows uniform dispersion of CNTs within the polymer matrix. The absence of voids or cracks suggests better percolation and matrix–filler compatibility at this concentration. The morphology suggests the presence of distinct CNTs rather than large agglomerates, which is generally desirable for reinforcing composites. Fig. 4f shows that moderate rGO loading (0.50 wt%) results in visible embedded CNCs and roughened fracture zones. The surface morphology appears more cohesive than 0.25 wt% rGO, which suggests improved filler–matrix interaction at this nanofiller concentration. Higher rGO content results in more compact fracture features but retains occasional voids, which is possibly due to local agglomeration or incomplete resin infiltration (Fig. 4g). However, CNC contributes to uniform filler dispersion and suppresses crack proliferation. Overall, the SEM analysis demonstrates that low concentrations of nanofillers, *i.e.* CNT and rGO, disperse well in the polymer matrix; higher loadings of nanofillers lead to increased agglomeration and poor interfacial adhesion.

**Fig. 5:**
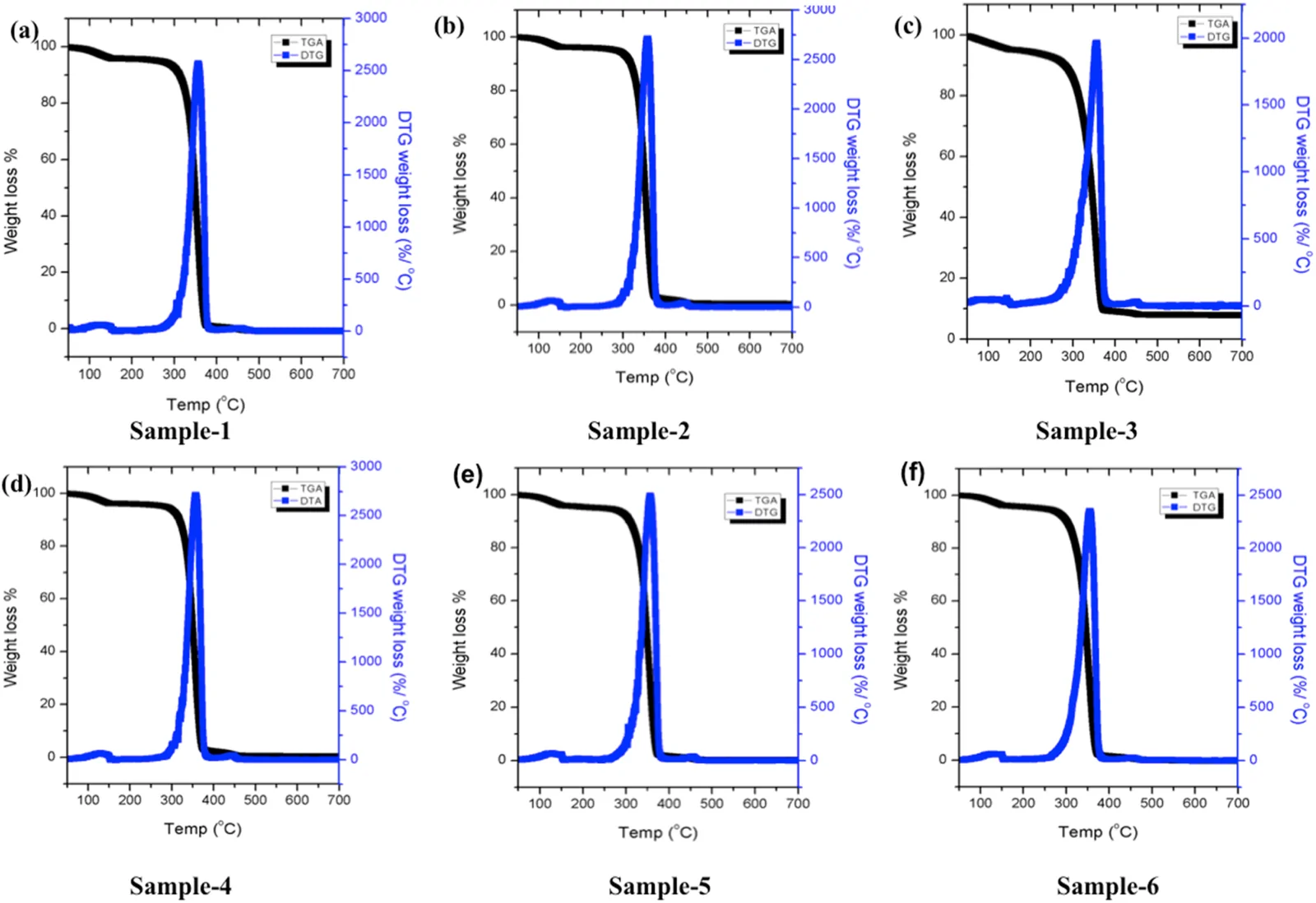
Thermogravimetric (TGA) and derivative (DTG) thermograms of PLA/CNC and nanocomposites. (a) Sample-1 (PLA + 1 wt% CNC), (b) sample-2 (0.25 wt% CNT), (c) sample-3 (0.25 wt% rGO), (d) sample-4 (0.50 wt% CNT), (e) sample-5 (0.50 wt% rGO), and (f) sample-6 (1 wt% rGO).

Under N_2_ atmosphere, the thermal stability of PLA/CNC composites was examined through thermogravimetric analysis (TGA) in order to determine the impact of CNT and rGO incorporation. Fig. 5 shows the related TGA and DTG curves, which indicate the weight loss as a function of temperature and the rate of weight loss. All samples exhibit a single, sharp degradation step between approximately 300 °C and 400 °C as reported in Table 3. This corresponds to the thermal decomposition of the PLA polymer backbone. The initial minor weight loss observed below 150 °C is attributed to the evaporation of residual moisture. The DTG peak in Fig. 5a indicates that the reference film has an onset of thermal degradation at approximately 320 °C, with the maximal rate of degradation (*T*_max_) occurring at approximately 354 °C. Almost complete degradation of organic components is confirmed by the relatively low residue. For sample 2, a slight shift in the onset and peak degradation temperatures is observed, as shown in Fig. 5b. The DTG peak broadens and shifts marginally to 360 °C, suggesting an improved thermal barrier effect due to the well-dispersed CNTs. The increase in thermal stability is attributed to the CNTs restricting polymer chain mobility and hindering thermal degradation pathways. Similar to CNTs, the incorporation of 0.25 wt% rGO causes a modest improvement in thermal resistance, with the DTG peak centered at 362 °C in Fig. 5c. The planar geometry and high thermal conductivity of rGO nanosheets likely aid in dissipating heat and delaying the degradation onset. As the concentration of rGO increases from 0.25 wt% (*T*_max_ = 362 °C) to 0.50 wt% (Fig. 5e, *T*_max_ = 369 °C), the thermal stability shows a marked improvement. A further increase to 1 wt% (Fig. 5f, *T*_max_ = 373 °C) results in an increase in the peak stability and remains significantly higher than the reference (sample 1). Additionally, a higher residual mass further supports enhanced thermal resistance. Increasing CNT content to 0.50 wt% (sample 4) further enhances thermal stability, with the DTG peak shifting to 368 °C (Fig. 5d). The more pronounced residue mass (4%) indicates higher thermal resistance, which may result from the formation of a protective carbonaceous layer that slows down volatilization. The TGA–DTG analysis confirms that incorporating CNTs and rGO is an effective strategy for enhancing the thermal stability of PLA/CNC nanocomposites. The nanofillers delay the onset of decomposition and shift the maximum degradation temperature to higher values. rGO was found to be a more effective thermal stabilizer than CNTs, likely due to its 2D barrier-forming capability.

**Table 3:** Thermal properties of PLA/CNC nanocomposites containing varying concentrations of CNT and rGO nanofillers

Sample	*T*_onset_ (°C)	*T*_max_ (°C)	Residual mass (wt%)
Sample 1	320	354	0.5
Sample 2	325	360	1.1
Sample 3	323	362	1.5
Sample 4	328	368	4.0
Sample 5	330	369	3.5
Sample 6	335	373	4.5

**Fig. 6:**
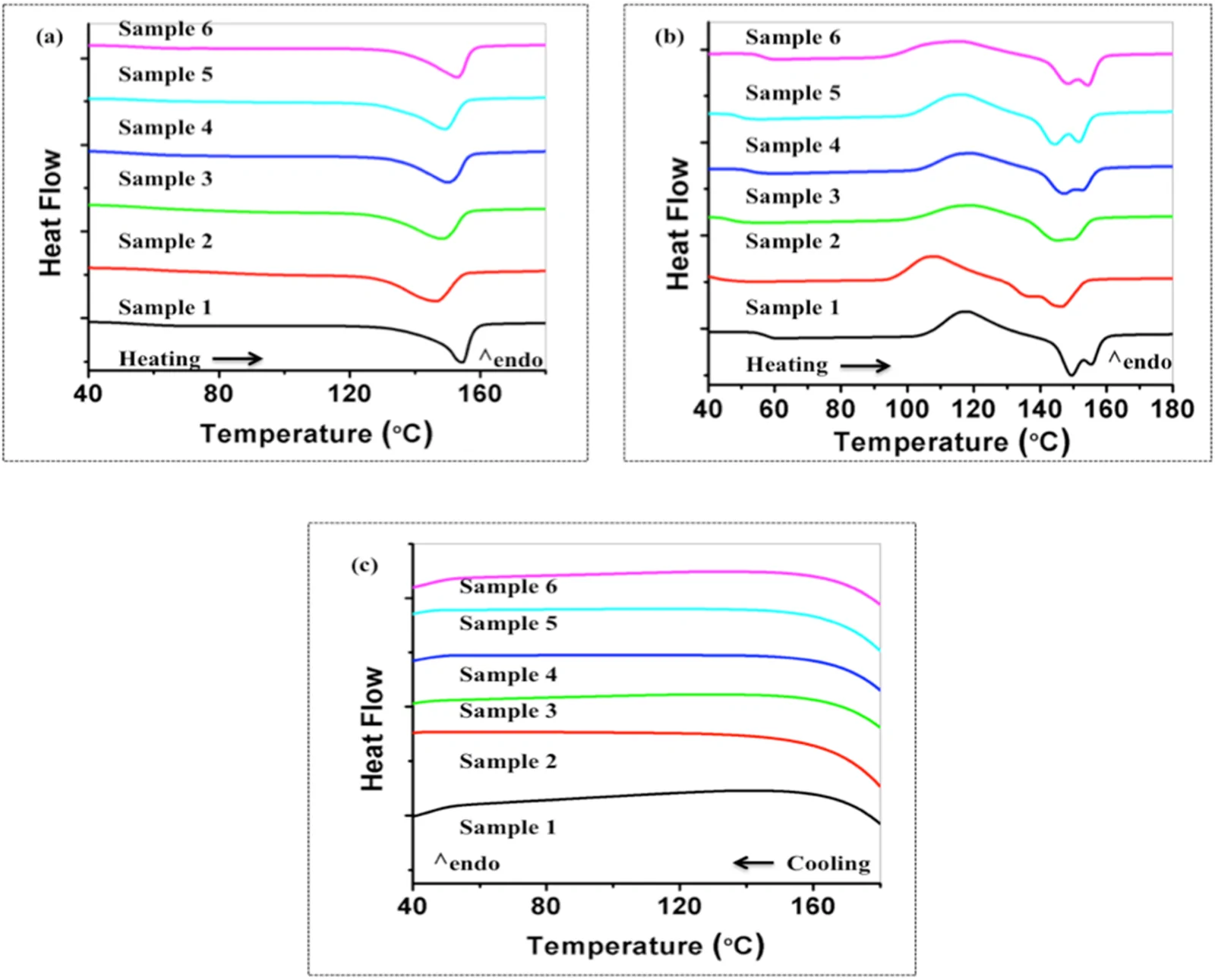
DSC spectra for PLA/CNC nanocomposites containing varying concentrations of CNT and rGO nanofillers: (a) DSC spectra for first heating, (b) second heating, and (c) first cooling for all nanocomposites.

It is essential to perform a heat–cool–heat cycle when conducting DSC analysis for polymeric materials, particularly semi-crystalline ones such as PLA. The prior thermal history of the sample was removed during the first cycle, and then the sample was subjected to controlled cooling and reheating settings to observe its behavior. The second cycle gave comprehensive information about glass transition, non-isothermal crystallization, and melting behaviour of nanocomposites to study the impact of nanofillers within the PLA matrix. Fig. 6a shows the DSC curve for the first heating, and Fig. 6b shows the DSC curve after the second heating for the PLA/CNC nanocomposites prepared by the solvent casting method. Glass transition temp (*T*_g_) was found to be 62.1 °C for sample 1. The addition of nanofillers did not result in any substantial changes to *T*_g_. At low loadings (samples 2–5), nanofillers (rGO/CNT) can restrict the segmental motion of PLA chains at the interface, thereby influencing *T*_g_ to a small extent, as nanofillers are well-dispersed and have strong interactions with the polymer, which can physically inhibit chain mobility. *T*_g_ for samples 2, 3, 4, and 5 are found to be 62.4, 62.4, 62.0, and 62.2 °C, respectively. Since nanofillers caused less relaxation of the amorphous portion of PLA, no endothermic peak for *T*_cc_ or enthalpy relaxation is observed, as shown in Fig. 6a, which indicates that the crystallization process has been completed before the measurement. Table 4 provides data on the thermal parameters and overall degrees of crystallinity obtained from the shown DSC scans. The addition of nanofiller, *i.e.*, rGO/CNT, didn't affect the cold crystallization too much but increased the melting enthalpy as well as crystallinity, as compared to the PLA + 1% CNC (sample 1) composite. Crystallinity increased from 22.6 (without nanofiller) to 30.2 with 0.5 wt% of CNT as nanofiller.

**Table 4:** Summary of the thermal properties of PLA/CNC/rGO/CNT nanocomposites obtained from DSC analysis

Sample name	First heating						Second heating						
	*T*_g_ (°C)	*T*_cc_ (°C)	*T*_m_ (°C)	Δ*H*_cc_ (J g^−1^)	Δ*H*_m_ (J g^−1^)	% *χ*_c_	*T*_g_ (°C)	*T*_cc_ (^o^C)	*T*_m_ (°C)		Δ*H*_cc_ (J g^−1^)	Δ*H*_m_ (J g^−1^)	% *χ*_c_
									*T*_m1_	*T*_m2_			
Sample 1	62.1	120.4	153.7	23.4	22.7	22.6	60.2	120.4	149.5	158.2	20.2	25.4	23.8
Sample 2	62.4	120.2	148.3	25.1	26.1	27.2	60.4	120.2	138.9	150.4	24.3	28.2	28.9
Sample 3	62.4	119.8	150.2	24.5	27.9	29.4	60.5	119.8	146.4	153.3	25.7	29.1	31.5
Sample 4	62.0	119.6	150.4	24.6	28.1	29.9	60.4	119.6	148.3	153.1	25.9	29.7	33.8
Sample 5	62.2	120.1	150.5	26.1	27.4	30.2	60.6	120.1	146.8	151.5	25.5	28.8	34.1
Sample 6	61.7	120.5	152.6	26.2	29.3	29.4	59.9	120.5	149.2	157.4	27.7	30.8	32.2

In the first cooling curves in Fig. 6c, all nanocomposites display an exothermic crystallization peak from the melt (*T*_c_). Notably, this peak is shifted to higher temperatures for the CNT- and rGO-filled samples (samples 2–6) compared to the pure PLA/CNC reference. This upward shift in *T*_c_ provides compelling evidence that both CNTs and rGO particles act as powerful nucleating agents, facilitating the formation of crystalline domains more efficiently and at an elevated temperature. This is a crucial finding, indicating improved processability and control over the final morphology of the composites. The DSC scan for second heating exhibit two distinct endothermic peaks (*T*_m1_ and *T*_m2_) as a result of the presence of both α and α′ polymorphs, as they melt away at different temperatures (Fig. 6b). It is possible that the melting of the α′ crystals, which are less stable, would correspond to the lower temperature peak *T*_m1_, while the α crystals, which are more stable, would correspond to the higher temperature peak *T*_m2_. Sometimes, the solid-state transition of α′ to α without total melting can be responsible for an exothermic peak prior to *T*_m1_. This behavior is typical of semi-crystalline PLA with CNC and rGO/CNT, which are working as heterogeneous nucleating agents. The crystallization behavior of the PLA matrix is significantly influenced by both rGO and CNTs. The steady rise in crystallinity (% *χ*_c_) from sample 1 to sample 6 (22.6% to 34.1%) reflects this nucleating capacity, most likely corresponding to increasing nanofiller concentration. During the second heating, a little decrement in *T*_g_ suggests molecular relaxation and a thermal history reset from residual stress release or chain rearrangement upon cooling. Δ*H*_m_ is greater than Δ*H*_cc_ for all samples, which confirms that a greater number of crystalline domains were present before reheating. This is likely a result of pre-crystallization induced by the nanofiller. The DSC results clearly indicate that both rGO and CNT improve the thermal properties and crystallinity of PLA/1 wt% CNC composites. Nonetheless, rGO demonstrates a remarkable improvement due to its 2D sheet structure, ability for interfacial bonding, and efficiency in nucleation.

The crystallization behaviour can also be interpreted in terms of established polymer crystallization models, such as the Johnson–Mehl–Avrami (JMA) approach for isothermal crystallization and the Ozawa and Liu-Mo models for non-isothermal crystallization.^39,40^ In the present PLA/CNC/rGO-CNT system, the increase in crystallization temperature and degree of crystallinity observed by DSC, together with the structural changes detected by XRD, the filler–matrix interactions identified by FTIR, and the filler dispersion and morphological features observed by FESEM, collectively indicate that rGO/CNT provides heterogeneous nucleation sites and influences the mobility and organization of PLA chains during crystallization.

### 3.2 *In vitro* lysozyme biodegradation

**Fig. 7:**
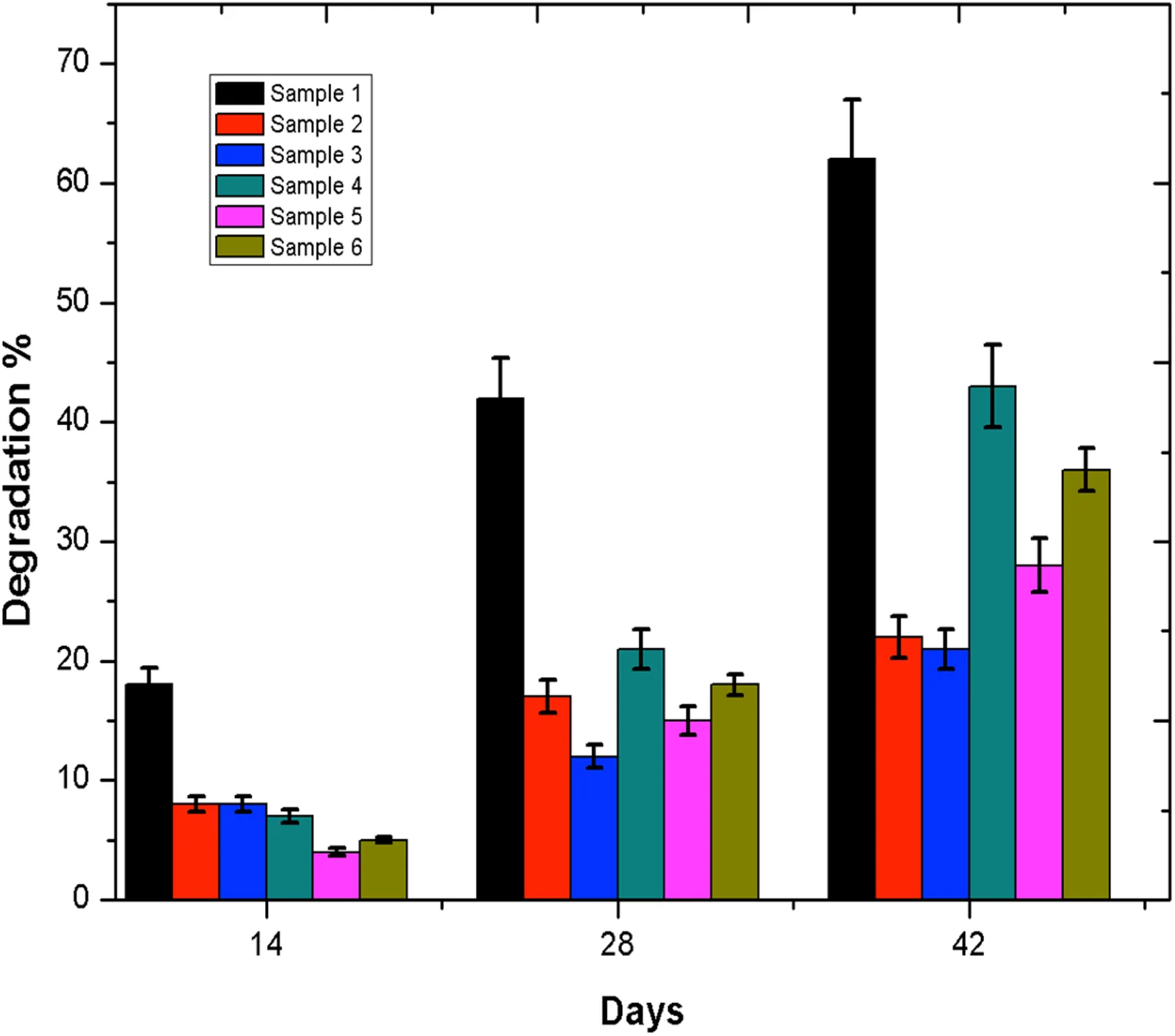
*In vitro* lysozyme-mediated biodegradation of PLA/CNC nanocomposites with varying CNT and rGO content over 42 days.

The enzymatic degradation of PLA/CNC nanocomposites, reinforced with varying concentrations of CNTs and rGO, was assessed over 42 days using lysozyme. Fig. 7 illustrates the substantial impact of nanofiller type and concentration on the degradation profile of the nanocomposites. Exposure to a lysozyme solution was employed to monitor the degradation of the films by measuring the percentage of mass loss over time. The reference composite (sample 1), which included PLA and 1 wt% CNC, demonstrated the fastest degradation, achieving more than 60% mass loss in just 42 days. This fast degradation is due to the hydrophilic properties of the CNCs, which enable the absorption of water and simultaneously promote hydrolytic cleavage of the PLA ester bonds. Consequently, the polymer matrix becomes more susceptible to enzymatic attack. The degradation rates in samples 2 and 3, which contained 0.25 wt% of CNT and rGO, were much lower (∼22% and ∼21%, respectively), emphasizing the barrier effects caused by the addition of carbon nanofillers. Lysozyme diffusion and enzymatic access to the ester bonds in PLA are restricted by the more compact structure that both CNT and rGO contribute to the matrix. Sample 4 (0.5 wt% CNT) exhibited a slight increase in degradation (∼27%) when compared to the 0.25 wt% CNT (sample 2). This may be attributed to partial CNT agglomeration at higher loading, which could produce localized weak zones that promote enzyme penetration. However, sample 5 (0.5 wt% rGO) exhibited a higher degree of degradation (∼35%) than all CNT-containing samples, indicating that rGO's 2-D morphology and improved dispersion facilitate partial moisture diffusion and lysozyme access. The degradation of rGO-containing films was observed to increase in the following order: 0.25 wt% (sample 3) <0.50 wt% (sample 5) < 1 wt% (sample 6). While the nanofillers inherently enhance barrier properties, this behavior suggests that they may begin to agglomerate at higher concentrations. This nanoparticle aggregation has the potential to introduce interfacial holes and defects within the PLA matrix, thereby compromising its structural strength and creating preferential channels for enzyme infiltration. Thus, degradation can be increased in comparison to the more optimally dispersed lower concentrations. These findings suggest that the degradability of the PLA/CNC matrix is altered by the incorporation of carbon nanofillers, particularly rGO, whereas CNCs increase enzymatic degradation through improved hydrophilicity.

### 3.3 Contact angle measurement

**Fig. 8:**
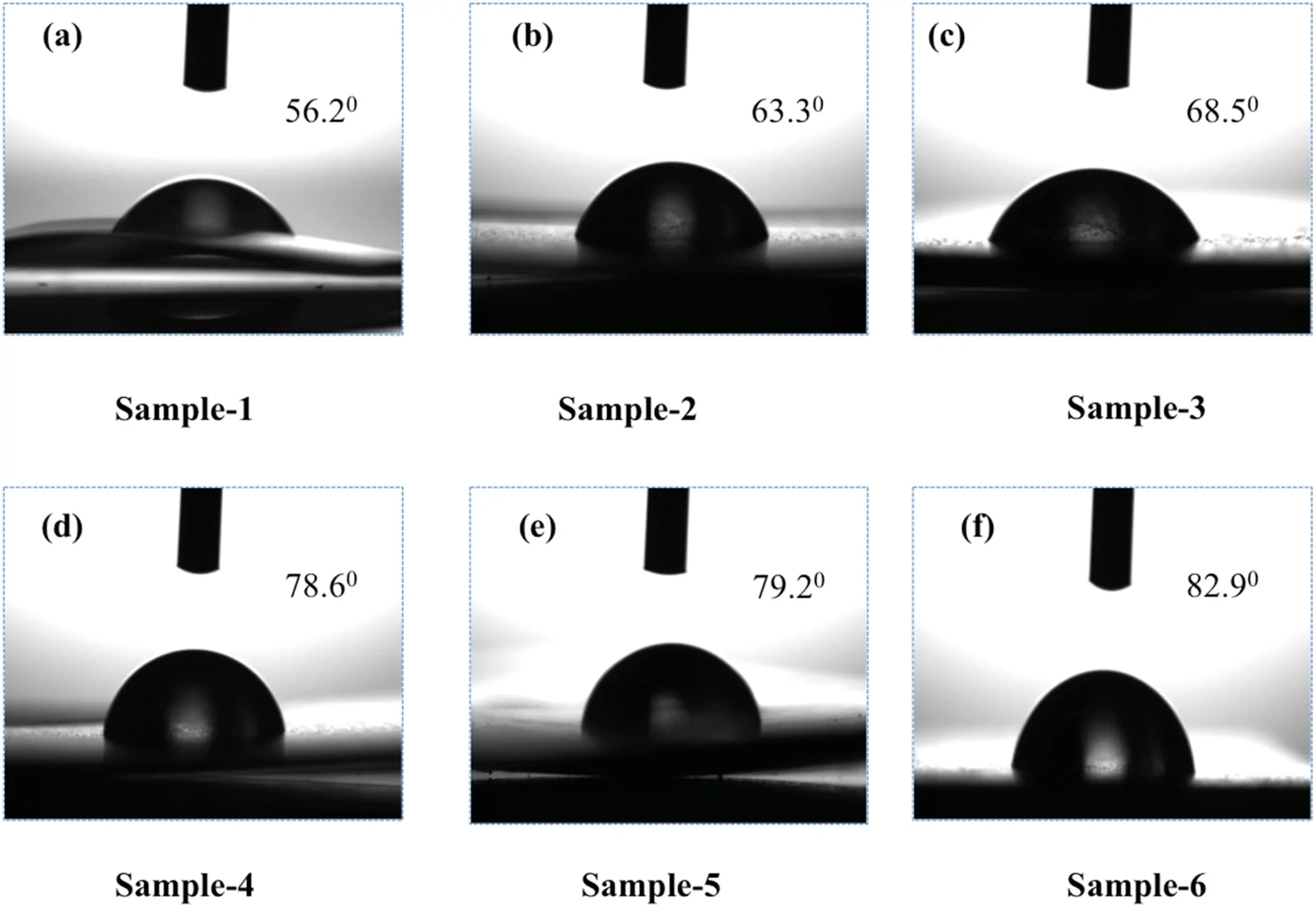
Water contact angle images of PLA/CNC-based nanocomposite films with varying CNT and rGO loadings. (a) Sample-1 (PLA + 1 wt% CNC), (b) sample-2 (0.25 wt% CNT), (c) sample-3 (0.25 wt% rGO), (d) sample-4 (0.50 wt% CNT), (e) sample-5 (0.50 wt% rGO), and (f) sample-6 (1 wt% rGO).

Surface wettability was assessed *via* the static sessile drop method. A prominent hydrophilic characteristic of CNCs is the abundance of hydroxyl (–OH) groups on their surface. CNCs are well-dispersed and have the potential to migrate to the surface or expose their hydrophilic –OH groups in the PLA matrix, thereby reducing the contact angle and turning the composite more hydrophilic than pristine PLA. The contact angle for sample 1 was reduced to 56.2° than 77° of pristine PLA, as reported.^41^ The rGO is relatively hydrophobic, consistent with the reduction of oxygen-containing functional groups during the hydrazine-assisted reduction process. The addition of rGO as a nanofiller makes the composite more hydrophobic. At low concentrations (samples 2 and 4), rGO might not have a significant impact on the CNC and PLA's predominant surface chemistry. Nevertheless, the contact angle experiences a modest increase as its hydrophobic characteristics begin to appear. Contact angles for samples 2 and 4 are 63.3° and 78.6°, respectively, as shown in Fig. 8. CNTs have graphitic non-polar surfaces, making them intrinsically hydrophobic materials. Their pristine surfaces have few polar functional groups that might interact with water. Their hydrophobic nature becomes increasingly dominant as the concentration of CNTs increases. Well-dispersed CNTs enhance the non-polar surface area, which reduces their favorable conditions for water spreading. Because CNTs have a high aspect ratio, even a small percentage of their weight can cover a substantial amount of surface area or impact the morphology of the surface. For sample 2, the contact angle increased to 68.5° and up to 79.2° for sample 5 as the CNT concentration increased to 0.5 wt% in the PLA/CNC composite. The wettability is significantly reduced as a result of the increased concentration of rGO (sample 6), and the contact angle is found 82.9°. This is because of the presence of rGO agglomeration in the composite. The Cassie–Baxter state suggests that surface roughness caused by rGO agglomeration may also be a factor in the appearance of high apparent contact angles.^42^

The absence of new absorption bands or significant peak shifts indicates that CNT and rGO are incorporated into the PLA/CNC matrix without forming new covalent bonds. The observed broadening and slight shift of the carbonyl band, particularly at higher rGO loading, suggest changes in the local polymer environment and matrix–nanofiller interactions. These interactions are therefore attributed primarily to noncovalent forces, including van der Waals interactions and hydrogen bonding involving the polar groups of PLA/CNC and residual oxygen-containing groups on rGO. Together with the SEM and XRD results, these observations support effective physical incorporation and dispersion of the nanofillers within the PLA/CNC matrix.

### 3.4 Cytocompatibility assessment (Alamar blue assay)

**Fig. 9:**
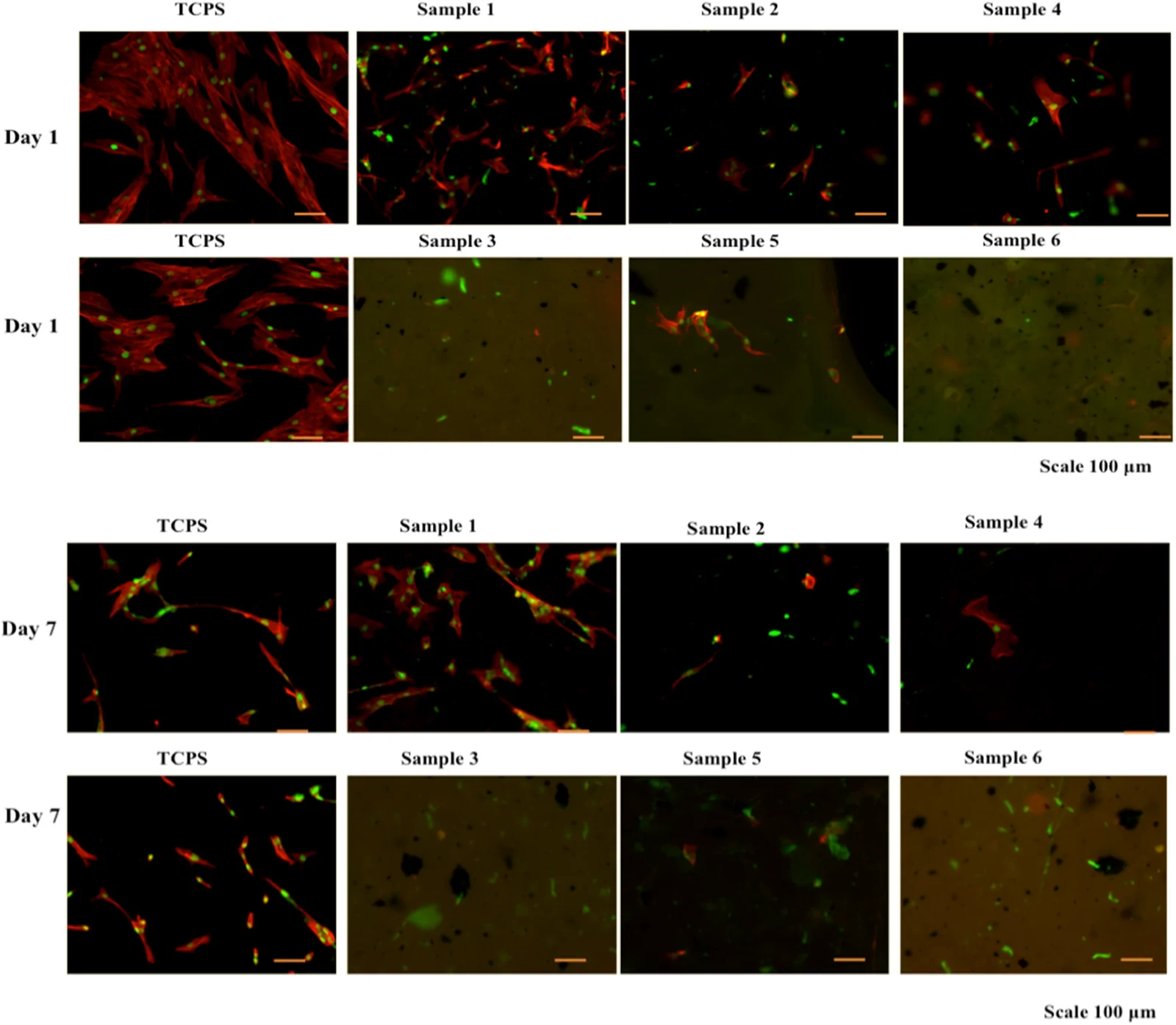
Live/dead staining of hMSCs cultured on nanocomposite films at day 1 and day 7. Live cells fluoresce green; dead cells fluoresce red, with fluorescence intensity (ex/em 560/590 nm).

**Fig. 10:**
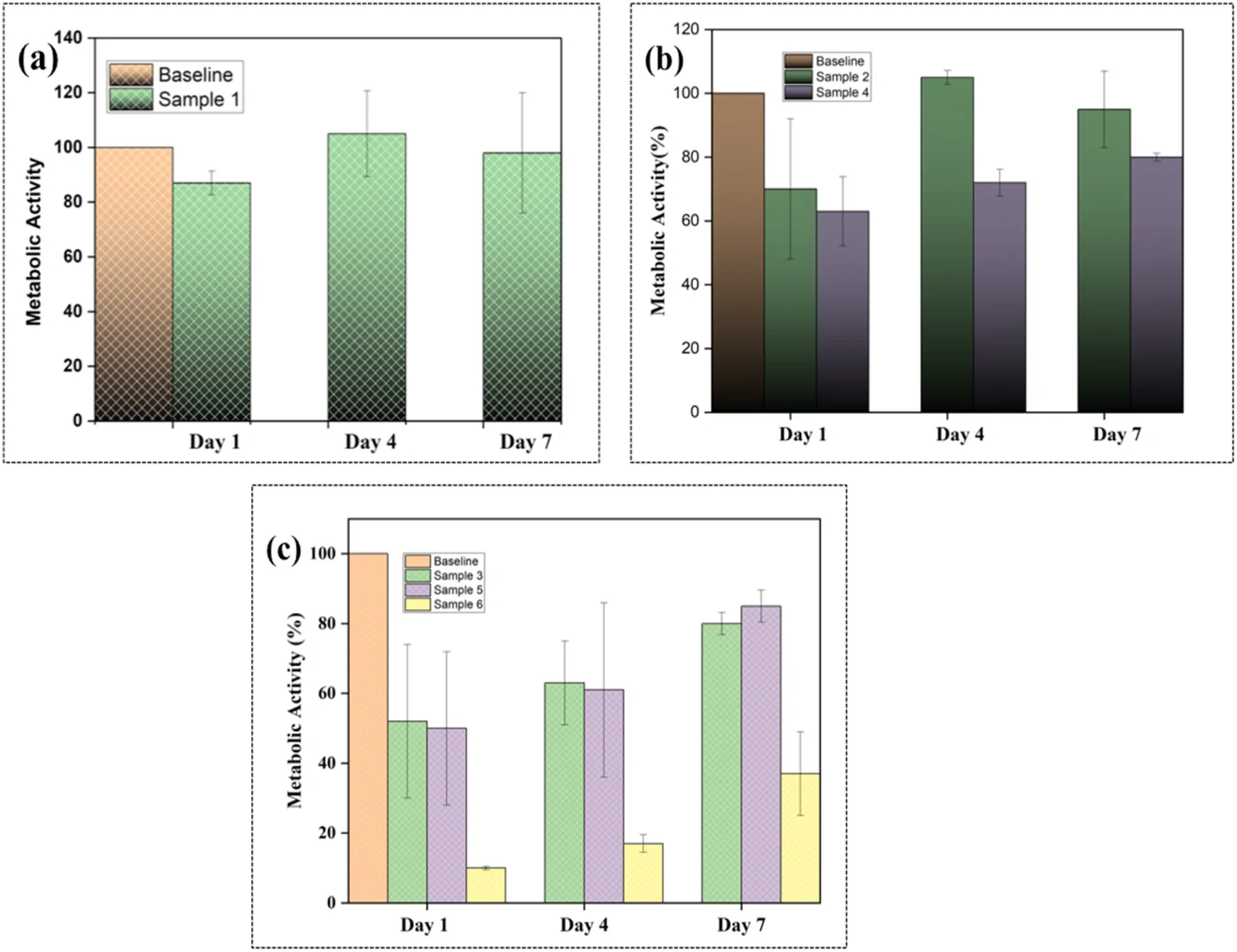
Alamar blue assay for hMSCs cultured on PLA/CNC films with different CNT and rGO loadings, relative to TCPS: (a) shows metabolic activity of PLA + 1 wt% CNC, (b) representation of the effect of CNC loading on metabolic activity, and (c) effect of rGO loading on metabolic activity relative to TCPS. Data shown as mean ± SD (*n* = 3). Statistical significance *versus* TCPS: *p* < 0.05, *p* < 0.01, *p* < 0.001.

To determine the efficacy of the developed scaffolds for supporting cell growth, the metabolic activity and morphology of human mesenchymal stem cells (hMSCs) were assessed using the Alamar blue assay over 7 days. The performance of PLA/CNC scaffolds modified with CNT and rGO was evaluated against the Tissue Culture Polystyrene (TCPS) negative control, with all quantitative data normalized to day 1 TCPS fluorescence. Fig. 10 Shows that dense green fluorescence and minimal red staining were observed in uniform, elongated hMSCs, which confirmed their outstanding viability and cell–substrate interaction. Sample 1 exhibited a slightly reduced day 1 activity (∼87% of TCPS), which was followed by a comparable growth trend to TCPS by day 7 (*p* > 0.05). This indicates that PLA/CNC is cytocompatible and has no significant impact on proliferation. On day 7, the fluorescence image of sample 1 (Fig. 9) showed that the cells were evenly distributed and exhibited a strong green fluorescence. However, the density of the cells was slightly lower than that of TCPS, which is consistent with the modest delay in proliferation that was observed in the Alamar blue assay. Metabolic activity in sample 2 was initially lower (∼70% of TCPS on day 1), but by day 7, there was a slight recovery (∼82% of TCPS). On the other hand, variable results were shown by high error bars, likely caused by inconsistent CNT dispersion. Partial CNT aggregation and the intrinsic hydrophobicity of CNTs, which influence protein adsorption and early adhesion, are held responsible for the inadequate cell-material interaction. On day 1, a few green-stained cells were observed, exhibiting minimal spreading. Despite a modest improvement in viability by day 7, it remained heterogeneous, with a moderate increase in fluorescence intensity. As CNT loading increased (sample 4), there was a more significant decrease in metabolic activity, as indicated by statistical significance (*p* < 0.01). On day 7, a slight upward trend was observed; however, the overall values remained substantially below the TCPS reference, indicating that the cytotoxicity from higher CNT loading increased. Very few adherent cells were observed. Red fluorescence, which indicates cytotoxicity, dominated in the majority of epifluorescence images (Fig. 9). The live cell density remained low even on day 7, confirming the reduced metabolic activity. Initial day 1 metabolic activity was approximately 45% of TCPS for sample 3, but by day 7, it exceeded up to 80% (*p* < 0.05) (Fig. 10). For sample 5, a small amount of viable cells were observed, but most had a spherical or poorly dispersed morphology. Increased red fluorescence and fragmented morphology indicate early-stage cellular death. The data support a modest but limited metabolic activity recovery. A significant reduction in activity was observed on day 1 (∼50% of TCPS), with only a small recovery by day 7 (∼85% of TCPS). The response implies a threshold concentration at which rGO starts to show cytotoxic or anti-proliferative effects, possibly as a result of ROS (reactive oxygen species) production, surface roughness, or cell death from sharp edges.^43^ Sample 6 showed the poorest performance. Metabolic activity was below 37% of TCPS at all times (*p* < 0.001), indicating sustained cytotoxicity (Fig. 10). This confirms the severe cytotoxic nature of high rGO content, as fluorescence microscopy revealed extensive cell death with minimal green signal and dominant red fluorescence even at day 7.

The combined use of quantitative metabolic activity and qualitative live/dead imaging allows a comprehensive understanding of the cell-material interface in these nanocomposites. CNC enhances biocompatibility, whereas CNT and rGO show dose-dependent effects; low concentrations keep acceptable cytocompatibility, while higher concentrations result in cytotoxic stress. It turns out that with a 0.25 wt% concentration of CNT, PLA/CNC is the most promising material. It is likely that its favorable physicochemical surface properties and negligible cytotoxicity are responsible for its capacity to enable greater hMSC adhesion, viability, and proliferation over time.

### 3.5 Water vapor permeability (WVP) measurements

**Fig. 11:**
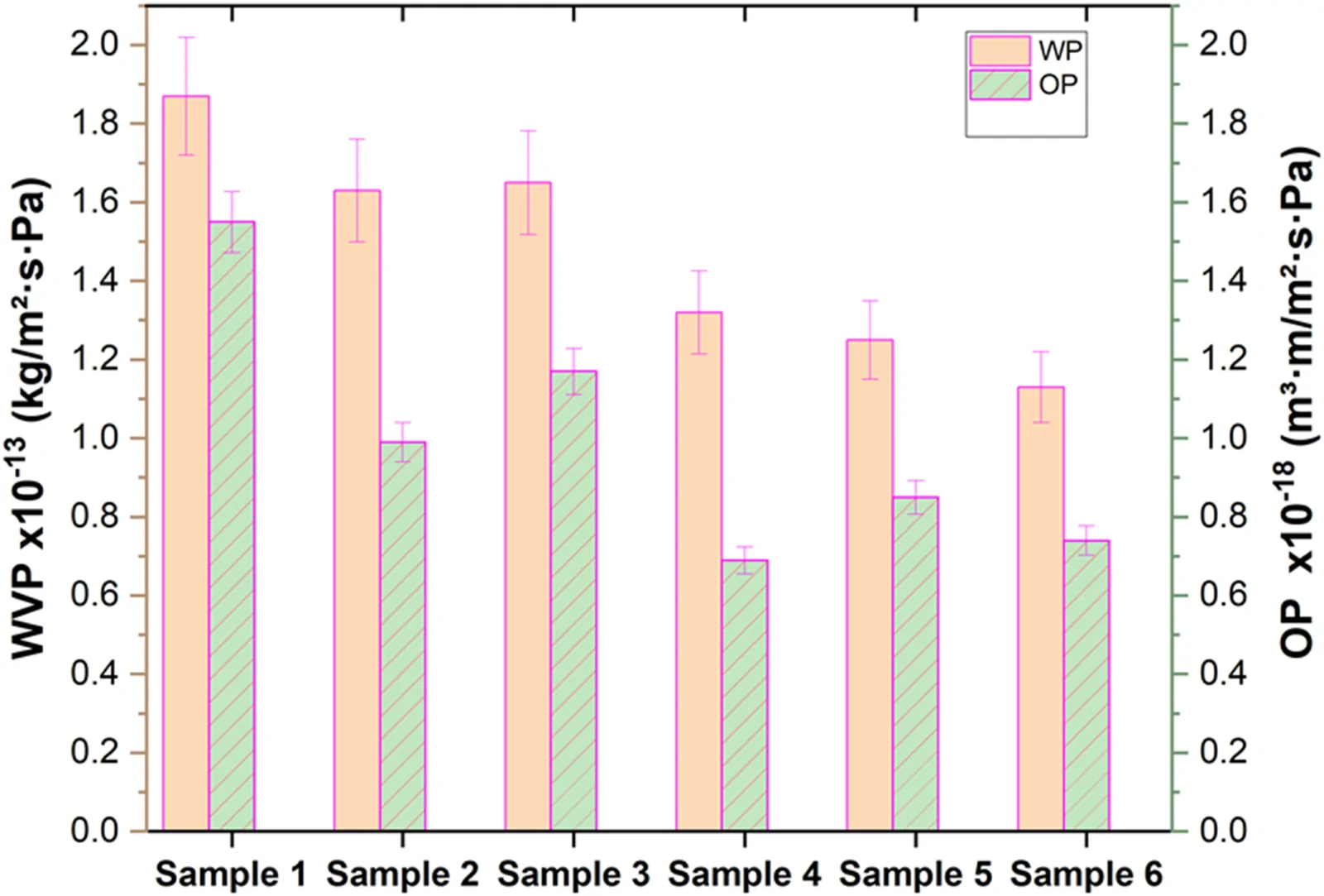
WVP and OP values of PLA/CNC-based nanocomposite films with varying CNT and rGO concentrations.

In the process of selecting materials for use in packaging applications, the WVP qualities are among the most important properties to consider. An important factor affecting packaged food is the amount of moisture that permeates the various materials used for packaging films. In order to prevent moisture from penetrating packaged food and causing damage to its quality, a barrier or wall is required in food packaging applications. Since the wall or barrier layer keeps the food moist and prevents it from drying out, the moisture barrier is very crucial when it comes to freshly packed food. The inherent semi-crystalline structure and amorphous regions of neat PLA enable the relatively simple diffusion of water vapor molecules, resulting in the highest WVP.^38^ For PLA crystallization, CNCs can function as nucleating agents, resulting in a greater degree of crystallinity. In general, crystalline regions are more impermeable than amorphous regions. Water vapor diffusion can be further restricted by strong hydrogen bonding between CNC and PLA chains, which can limit polymer chain mobility and decrease free volume. Table 5 shows the water vapor barrier properties for all the samples. WVP for sample 1 (PLA + 1% CNC) is 1.87 ± 0.09, and this is taken as the baseline for other composites (Fig. 11). When 0.25 wt% CNT was incorporated into the PLA/CNC composite (sample 2), the WVP decreased by 13%, with a measured value of 1.63 ± 0.13. This reduction can be attributed to the presence of well-dispersed CNTs, which introduce additional barrier effects and increase the tortuosity of diffusion pathways within the PLA/CNC matrix. WVP for sample 3 is similar to sample 2, as rGO increased the crystallinity of the composite. The increased crystallinity may be attributed to the improved dispersion of rGO into the polymer matrix, which led to a decrease in permeability by decreasing the cross-sectional surface for diffusion and raising the path length of diffusion. For samples 4 and 5, the WVP was further decreased, which may be attributed to the well-dispersed and mixed effect of CNT and rGO in the PLA/CNC matrix. The sheet-like structure of rGO was a little more effective than CNTs in preventing the motion of vapor, which indicates that it has a superior lamellar barrier effect when it is well-dispersed.^32^ WVP was reduced to a maximum of 40% for sample 6 in comparison to sample 1 when rGO loading is maximum (1 wt%). These results are consistent with the DSC and wettability results; as crystallinity and hydrophobicity increased, the % WVP reduction followed the same pattern.

**Table 5:** WVP and OP of PLA/CNC and rGO/CNT modified samples

Samples	WVTR × 10^−6^ (kg m^−2^ s^−1^)	WVP × 10^−13^ (kg m^−2^ s^−1^ Pa)	% WVP Reduction	OP ×10^−18^ (m^3^ m m^−2^ s^−1^ Pa)	% OP Reduction
Sample 1	1.50 ± 0.04	1.87 ± 0.09	—	1.55 ± 0.16	—
Sample 2	1.29 ± 0.10	1.63 ± 0.13	13	0.99 ± 0.21	26
Sample 3	1.32 ± 0.07	1.65 ± 0.21	12	1.17 ± 0.08	24
Sample 4	1.04 ± 0.05	1.32 ± 0.16	30	0.69 ± 0.19	55
Sample 5	0.99 ± 0.09	1.25 ± 0.11	33	0.85 ± 0.25	45
Sample 6	0.90 ± 0.13	1.13 ± 0.05	40	0.74 ± 0.14	52

### 3.6 Oxygen permeability (OP) measurements

The impact of varying concentrations of rGO and CNT on the OP of a baseline PLA + 1% CNC composite is presented in Table 5. Sample 1 exhibited an OP of 1.55 ± 0.16 × 10^−18^ m^3^ m m^−2^ s^−1^ Pa, which is better than pristine PLA, according to the reported literature.^38^ The OP decreased significantly with both rGO and CNT in a concentration-dependent way. The OP consistently decreased as the nanofiller dosage increased from 0.25 wt% to 1.00 wt% (Fig. 11), showing the improvement of barrier performance. CNTs consistently reduced OP more than rGO at the same weight percentage. At 0.25 wt% loading of nanofiller, CNT achieved a better reduction of 26% (OP 0.99 ± 0.21) than rGO's OP (1.17 ± 0.08) of 24%. The increased efficacy of CNTs may suggest that, when well-dispersed, their 1D thread-like structure may provide effective resistance to gas permeation when sufficiently dispersed than the more planar character of rGO at equivalent weight loadings, particularly if rGO undergoes some restacking. At 0.50 wt%, CNT (sample 4) showed a 55% reduction (OP = 0.69 ± 0.19) compared to rGO's (sample 5) 45% reduction (OP = 0.85 ± 0.25). Sample 6 showed a 52% reduction with OP = 0.74 ± 0.14. These results are consistent with the DSC, wettability, and WVP results, as crystallinity and hydrophobicity increased, the OP also decreased simultaneously. Using low concentrations of carbon-based nanofillers (rGO and CNT) within a PLA-CNC matrix, it is possible to reduce OP. This is in perfect alignment with the current emphasis on sustainable and advanced nanocomposites. At equivalent loadings, the superior performance of CNTs indicates an optimized design approach for the improvement of gas barrier properties in these nanocomposites.

The reduction in WVP and OP is consistent with increased diffusion resistance arising from the incorporation of CNT and rGO within the PLA/CNC matrix. The high-aspect-ratio CNTs and layered rGO can increase the effective path length for diffusing molecules when sufficiently dispersed.

## 4. Conclusion

In summary, PLA/CNC nanocomposite films containing CNT or rGO were prepared by solution casting and evaluated with respect to their structural, thermal, surface, barrier, degradation, and biological properties. XRD and DSC results showed increased PLA crystallinity after nanofiller incorporation, consistent with heterogeneous nucleation by CNT and rGO. The absence of a distinct graphitic diffraction feature suggests that pronounced CNT/rGO restacking was not evident in the composite films, while SEM provided complementary evidence of concentration-dependent filler dispersion. FTIR analysis indicated changes in the local chemical environment consistent with noncovalent matrix–nanofiller interactions without detectable formation of new covalent bonds.

The incorporation of CNT and rGO improved the thermal stability and reduced both water-vapor and oxygen permeability of the PLA/CNC matrix. These barrier improvements are consistent with increased resistance to molecular transport associated with the incorporation of high-aspect-ratio CNTs and layered rGO. The contact-angle measurements further demonstrated a systematic change in surface wettability with nanofiller incorporation. Biological testing showed that the response depended on filler type and concentration, with low CNT loading exhibiting comparatively favorable cytocompatibility, whereas higher rGO loading produced pronounced cytotoxic effects.

Lysozyme-assisted degradation confirmed that the nanocomposites undergo degradation under the controlled laboratory conditions used in this study. These results demonstrate that nanofiller type and concentration can be used to tune the structural, thermal, barrier, surface, and degradation properties of PLA/CNC composites. The developed films therefore show potential for sustainable packaging-related applications; however, food-contact migration, realistic storage stability, standardized environmental biodegradation/composability, film-thickness uniformity, and large-scale continuous processing require further investigation before practical deployment.

## Conflicts of interest

The authors confirm that no financial or personal relationships influenced this work. No dedicated funding was received from government, industry, or non-profit organizations.

## Supplementary Material

RA-OLF-D6RA05823J-s001

## Data Availability

The data that support the findings of this study are available from the corresponding author upon reasonable request. Supplementary information (SI) is available. See DOI: https://doi.org/10.1039/d6ra05823j.
