# Supplementary material for: Poly(lactic acid) hybrid nanocomposites with synergistic cellulose nanocrystal-carbon reinforcement for sustainable packaging and multifunctional applications
Source: RSC Adv. 2026 Sep 16;16(44):55434–48. doi: 10.1039/d6ra05823j (PMC13579534; doi:10.1039/d6ra05823j)
Supplement: Supplementary file 1 [file RA-016-D6RA05823J-s001.pdf]

Supplementary Information (SI) for RSC Advances.
This journal is © The Royal Society of Chemistry 2026

Supporting Information

Poly(lactic acid) Hybrid Nanocomposites with Synergistic Cellulose Nanocrystal–Carbon Reinforcement for Sustainable Packaging and Multifunctional Applications

Kartikey Verma [a], Salim H. Siddiki [b], Chandan Kumar Maity [c], Bhuvaneshwari Balasubramaniam [d], Anup Singh [e], Tiyasha Tiyasha [f], Suraj Kumar Bhagat [g], Ankush Mehta [h], and Jitendra Bahadur [i]*

[a] Krishna Institute of Engineering and Technology (KIET), Ghaziabad, Delhi-NCR, Uttar Pradesh, 201206, India.

[b] Department of Chemistry and Chemical Biology, IIT (ISM) Dhanbad, 826004, Dhanbad, India.

[c] Department of Chemistry, Gachon University, 1342 Seongnam-daero, Sujeong-gu, Seongnam-si, Gyeonggi-do 13120, Republic of Korea.

[d] Department of Chemistry, Indian Institute of Technology (BHU) Varanasi, Varanasi, Uttar Pradesh 221005, India.

[e] University Centre for Research & Development, Chandigarh University, Gharuan, 140413, Punjab, India.

[f] Faculty of Civil Engineering, Ton Duc Thang University, Ho Chi Minh City, 700000, Vietnam.

[g] Centre for Interdisciplinary Research, SRM University-AP, Amarawati, Andhra Pradesh 522240, India.

[h] School of Engineering and Technology, K.R. Mangalam University, Gurugram, 122103, Haryana, India.

[i] Electronics and Communication Engineering, Amrita School of Engineering, Amrita Vishwa Vidyapeetham, Bengaluru, 560103, Karnataka, India.

***Corresponding authors:** Suraj Kumar Bhagat (surajkumar.b@srmap.edu.in).

S1. Experimental section

S1.1 Materials details

The reagents used in the synthesis of rGO were obtained from Sigma Aldrich (India) with purity levels >99.9%. Graphite powder, orthophosphoric acid, HCl (hydrochloric acid 10%), , H_2SO_4, $KMnO_4$, and hydrogen peroxide (H_2O_2, 30%) are some of the reagents. MWCNT (purity > 98%, diameter 8.7 nm-10 nm & surface area -216m^2/g) was purchased from Sigma-Aldrich. Natur-Tec, India supplied Poly Lactic Acid (PLA 4044D) with glass transition Temp 55-60 °C for the study. Highly pure Chloroform, Ethanol were also obtained from Sigma Aldrich. Every solvent & reagent were used exactly as supplied.

S1.2 Synthesis of reduced graphene oxide (rGO)

GO (graphene oxide) was synthesized through a well-established Modified Hummer's method [36]. For the synthesis of rGO, GO was chemically reduced with hydrazine hydrate ($N_2H_4 \cdot H_2O$). As-prepared GO was usually sonicated for 30 minutes after being dissolved in deionized water for adequate exfoliation. Subsequently, $N_2H_4 \cdot H_2O$ was introduced into the GO dispersion. After that, a water-cooled condenser was used to heat the mixture under reflux for 24 hours at 100 ^{0}C. During this reduction process, the solution visibly transformed, becoming black with the formation of distinct rGO flakes. The obtained rGO dispersion was thoroughly washed with methanol, followed by repeated washing with deionized water to achieve neutral pH. The suspension was kept to settle for 24 hours, and then rGO was collected by filtration. The isolated rGO was subsequently desiccated in an oven at 50 ^{0}C and stored for future use.

S1.3 Material characterizations

FT-IR

FT-IR spectra were acquired using a Bruker Tensor 27 FT-IR Spectrometer. The resolution of each spectrum was 4 cm^{-1}, & 64 scans were averaged across the 4000-500 cm^{-1} wavenumber range. Prior to analysis, all samples were desiccated for 24 hours in an oven at 60 ^{0}C to ensure consistent data. Furthermore, sample thickness was precisely kept at a uniform level across all measurements.

XRD

XRD was carried out to analyse the crystalline structure and phase composition of PLA/CNC-based nanocomposites with varying concentrations of CNT and rGO. The study was conducted using a Panalytical XPert X-ray diffractometer operating with Cu Kα radiation (λ = 1.5406 Å) at 40 kV and 40 mA. The scanning range (2θ) of 5°–60° was scanned at a speed of 1°/min with a step size of 0.02°.

Scherrer equation was used to calculate the average crystallite size (D).

$$D = \frac{0.9\lambda}{\beta cos\theta}$$

where, Bragg's angle is θ and β is the FWHM (in radians) & λ is the x-ray wavelength.

Crystallinity Index (CI) is calculated by

$$CI(\%)\frac{I002 - Iam}{I002}x100$$

Where,

I_{002} is the crystalline peak at (002) plane, I_{am} is the intensity at 2θ.

SEM

10 kV of accelerating voltage and a working distance of 50 mm were utilized to perform surface morphological characterization using a Tescan MIRA3 scanning electron microscope. Prior to imaging, film samples were carefully sliced into small sizes and put onto carbon tape for conduction. To diminish the effects of charging and improve conductivity, the samples were cryo-treated in liquid nitrogen and subsequently gold-sputtered for 90 seconds using a sputter-coating apparatus.

TGA

The thermal degradation characteristics of each sample were performed using thermogravimetric analysis (TGA). In liquid nitrogen, the experimentation was conducted on a TA Instruments (DSC SDT Q600 Thermogravimetric Analyzer) at temperatures (25 -600 °C) at heating rate of 10 °C/min.

DSC

In order to assess the thermal characteristics, differential scanning calorimetry (DSC) was employed. A TA Instruments (DSC Q25) device with a 50 mL/min of N_2 purge rate, was used in the experimental setup. The samples weighed 10 mg and were equilibrated in the DSC at 10°C and being put into aluminum containers. Furthermore, the specimens were heated at a rate of 10°C/min, which ended at a maximal 200°C temperature. After that, a cooling process was conducted at 10°C/min rate until the 10°C initial temperature was reached. For the second heating and cooling cycle, the same procedure was carried out.

Wettability & contact angle measurement

Surface wettability of the PLA/CNC-based nanocomposite films was studied using a contact angle goniometer (KYOWA interface measurement system) via the sessile drop method. A droplet of distilled water (6 µL) was carefully placed on the film surface, and static contact angles were recorded after 60 seconds to ensure equilibrium. For statistical

robustness, measurements were performed at ten different locations on each film. The contact angle values were analyzed using ImageJ software equipped with a contact angle plugin to quantify the hydrophilicity variations introduced by CNT/rGO incorporation.

In Vitro enzymatic biodegradation test

The in-vitro enzymatic degradation behaviour of PLA/CNC and rGO/CNT loaded nanocomposite films was evaluated by lysozyme to simulate physiological enzymatic conditions. Neat PLA/CNC film (1 wt% CNC) was used as the reference. All films were cut into rectangular specimens (1 × 1 cm²), accurately weighed (initial dry weight, W_0), and sterilized via 70% ethanol immersion followed by UV exposure for 30 minutes on each side. 10 mL of phosphate-buffered saline (PBS, pH 7.4) were used to immersed the samples, containing 1.5 μg/mL lysozyme (Sigma-Aldrich) and samples were incubated at 35 °C under static conditions for up to 42 days. The enzyme solution was refreshed every 48 hours to maintain activity. At predetermined time intervals (Day 14, 28, and 42), samples were removed, thoroughly rinsed with distilled water to remove surface-adsorbed salts and enzymes, and dried or 48 hours under vacuum at 35 °C. Each degradation experiment was performed in triplicate. In vitro, the specimens were subjected to a pre-degradation weighing process (w_0) as well as a after-degradation weighing process (w_1). Utilizing the sample's weight loss in relation to its initial weight, the deterioration degree (Δw) was determined:

$$\Delta w(\%) = (\frac{w_0 - w_1}{w_0}) \times 100$$

Alamar blue assay

hMSCs (Human mesenchymal stem cells) procured from Sigma-Aldrich were employed to evaluate the cytocompatibility of PLA/CNC-based nanocomposite films using the Alamar Blue assay. 10% fetal bovine serum (FBS) and 1% penicillin-streptomycin were

added to Dulbecco's Modified Eagle's Medium (DMEM, high glucose), used to culture the cells. The cultivation was conducted under standard conditions (37°C, 5% CO_2). The process was followed to synthesize the nanocomposite films, which were then sterilized with 70% v/v ethanol and exposed to UV light for 30 minutes on each side. The sterilized films were then placed into the wells of a 24-well tissue culture plate, with tissue culture polystyrene (TCPS) serving as the control substrate. hMSCs were seeded onto each film and control wells at 1×10^4 cells/well density & incubated for up to 7 days. On days 1, 4, and 7, the medium was replaced with fresh medium containing 10% (v/v) Alamar Blue reagent (Thermo Fisher Scientific), and cells were incubated at 37 °C for 2h. The fluorescence was measured using a microplate reader (excitation: 560 nm, emission: 590 nm) after 200 μL of the medium was transferred to a 96-well plate after incubation.

Water vapor permeability (WVP)

WVP was determined in compliance with ASTM E96-80 by employing the gravimetric cup method (desiccant method) [37]. Small films were sealed over aluminum test cups containing 2 g of anhydrous $CaCl_2$ & placed in a humidity-controlled chamber kept at 75 % RH and 25°C. The increase in cup weight due to water vapor uptake was recorded at 12 h intervals over 3 days. WVP (water vapor permeability) & WVTR (water vapor transmission rate) was calculated using:

$$WVTR = \frac{\Delta m}{A.\Delta t}$$

Where, where Δm is the mass of water vapor transmitted (kg), A is the exposed film area (m^2), Δt is the exposure time (s).

$$WVP = \frac{WVTR.d}{\Delta P}$$

where, d is the average thickness of the film. Partial pressure difference (Pa) of water vapor across the film is ΔP. The final units of WVP was expressed as $kg/m^2\cdot s\cdot Pa$.

Oxygen permeability (OP)

OP was measured using a PMI-USA following ASTM D3985. Films were clamped in diffusion cells and exposed to high-purity oxygen (100%) on one side and nitrogen (carrier gas) on the other, at 23 °C and 0% RH isolate oxygen transmission. The oxygen transmission rate (OTR) was calculated and converted to OP using the following equation [37].

$$OP = \frac{0TR.d}{\Delta P}$$

where OTR is the oxygen transmission rate ($m^3/m^2 \cdot s$), d is the film thickness (m), & ΔP is the oxygen partial pressure difference (Pa). Final OP values are expressed in $m^3 \cdot m/m^2 \cdot s \cdot Pa$.

Statistical analysis

All data are expressed as mean ± standard error of the mean (SEM) from three independent experiments. Statistical significance was determined using ordinary one-way ANOVA followed by Sidak's multiple comparison test, performed in GraphPad Prism 6.01 (GraphPad Inc., CA, USA). Differences were considered statistically significant at $p < 0.05$.

Table 1: Sample code and the amount of different components used for PLA/CNC/CNT/rGO nanocomposites.

Sample code	PLA (wt%)	CNC (wt%)	CNT (wt%)	rGO (wt%)
**Sample 1**	99.0	1.0	-	-
**Sample 2**	98.75	1.0	0.25	-
**Sample 3**	98.75	1.0	-	0.25
**Sample 4**	98.5	1.0	0.50	-
**Sample 5**	98.5	1.0	-	0.50
**Sample 6**	98.0	1.0	-	1.0

Table 2: Structural parameters derived from XRD patterns for the PLA+CNC nanocomposites. Analysis focuses on the principal (200)/(110) reflection.

Sample	2θ (°)	*d*-Spacing (Å)	FWHM (°)	Relative Crystallinity Index (% CI)
**Sample 1**	16.62	5.29	0.89	29.6
**Sample 2**	16.59	5.27	0.81	32.9
**Sample 3**	16.63	5.27	0.83	37.2
**Sample 4**	16.61	5.28	0.73	36.6
**Sample 5**	16.65	5.30	0.75	40.4
**Sample 6**	16.6 3	5.30	0.72	44.1

Table 3: Thermal properties of PLA/CNC nanocomposites containing varying concentrations of CNT and rGO nanofillers.

Sample	T_{onset} (°C)	T_{max} (°C)	Residual Mass (wt%)
**Sample 1**	320	354	0.5

**Sample 2**	325	360	1.1
**Sample 3**	323	362	1.5
**Sample 4**	328	368	4.0
**Sample 5**	330	369	3.5
**Sample 6**	335	373	4.5

Table 4: Summary of the thermal properties of PLA/CNC/rGO/CNT nanocomposites obtained from DSC analysis.

**Sample Name**	**First Heating**						**Second Heating**						
	**T_g (°C)**	**T_{cc} (°C)**	**T_m (°C)**	**ΔH_{cc} (J/g)**	**ΔH_m (J/g)**	**%χc**	**T_g (°C)**	**T_{cc} (°C)**	**T_m (°C)**		**ΔH_{cc} (J/g)**	**ΔH_m (J/g)**	**%χc**
									**T_{m1}**	**T_{m2}**			
**Sample 1**	62.1	120.4	153.7	23.4	22.7	22.6	60.2	120.4	149.5	158.2	20.2	25.4	23.8
**Sample 2**	62.4	120.2	148.3	25.1	26.1	27.2	60.4	120.2	138.9	150.4	24.3	28.2	28.9
**Sample 3**	62.4	119.8	150.2	24.5	27.9	29.4	60.5	119.8	146.4	153.3	25.7	29.1	31.5
**Sample 4**	62.0	119.6	150.4	24.6	28.1	29.9	60.4	119.6	148.3	153.1	25.9	29.7	33.8
**Sample 5**	62.2	120.1	150.5	26.1	27.4	30.2	60.6	120.1	146.8	151.5	25.5	28.8	34.1
**Sample 6**	61.7	120.5	152.6	26.2	29.3	29.4	59.9	120.5	149.2	157.4	27.7	30.8	32.2

Table 5: Water vapor and oxygen permeability of PLA/CNC and rGO/CNT modified samples.

Samples	WVTR x 10^{-6} ($kg/m^2 \cdot s$)	WVP x10^{-13} ($kg/m^2 \cdot s \cdot Pa$)	%WVP Reduction	OP x10^{-18} ($m^3 \cdot m/m^2 \cdot s \cdot Pa$)	%OP Reduction
**Sample 1**	1.50± 0.04	1.87 ± 0.09	-	1.55± 0.16	-
**Sample 2**	1.29± 0.10	1.63± 0.13	13	0.99± 0.21	26
**Sample 3**	1.32± 0.07	1.65± 0.21	12	1.17± 0.08	24
**Sample 4**	1.04± 0.05	1.32± 0.16	30	0.69± 0.19	55
**Sample 5**	0.99± 0.09	1.25± 0.11	33	0.85± 0.25	45
**Sample 6**	0.90± 0.13	1.13± 0.05	40	0.74± 0.14	52
